# Influenza interaction with cocirculating pathogens and its impact on surveillance, pathogenesis, and epidemic profile: A key role for mathematical modelling

**DOI:** 10.1371/journal.ppat.1006770

**Published:** 2018-02-15

**Authors:** Lulla Opatowski, Marc Baguelin, Rosalind M. Eggo

**Affiliations:** 1 Université de Versailles Saint Quentin, Institut Pasteur, Inserm, Paris, France; 2 London School of Hygiene & Tropical Medicine, London, United Kingdom; 3 Public Health England, London, United Kingdom; University of Alberta, CANADA

## Abstract

Evidence is mounting that influenza virus interacts with other pathogens colonising or infecting the human respiratory tract. Taking into account interactions with other pathogens may be critical to determining the real influenza burden and the full impact of public health policies targeting influenza. This is particularly true for mathematical modelling studies, which have become critical in public health decision-making. Yet models usually focus on influenza virus acquisition and infection alone, thereby making broad oversimplifications of pathogen ecology. Herein, we report evidence of influenza virus interactions with bacteria and viruses and systematically review the modelling studies that have incorporated interactions.

Despite the many studies examining possible associations between influenza and *Streptococcus pneumoniae*, *Staphylococcus aureus*, *Haemophilus influenzae*, *Neisseria meningitidis*, respiratory syncytial virus (RSV), human rhinoviruses, human parainfluenza viruses, etc., very few mathematical models have integrated other pathogens alongside influenza. The notable exception is the pneumococcus–influenza interaction, for which several recent modelling studies demonstrate the power of dynamic modelling as an approach to test biological hypotheses on interaction mechanisms and estimate the strength of those interactions.

We explore how different interference mechanisms may lead to unexpected incidence trends and possible misinterpretation, and we illustrate the impact of interactions on public health surveillance using simple transmission models. We demonstrate that the development of multipathogen models is essential to assessing the true public health burden of influenza and that it is needed to help improve planning and evaluation of control measures. Finally, we identify the public health, surveillance, modelling, and biological challenges and propose avenues of research for the coming years.

## Author summary

Influenza is responsible for major morbidity and mortality burdens worldwide. Mathematical models of influenza virus transmission have been critical to understanding the virus epidemiology and planning public health strategies for infection control. It is increasingly clear that microbes do not act in isolation but potentially interact within the host. Therefore, studying influenza alone may lead to misinterpretation of transmission or severity patterns. Here, we review the literature on bacteria and viruses that interact with influenza, proposed interaction mechanisms, and mathematical modelling studies that include interactions. We report evidence that, beyond the classic secondary bacterial infections, many pathogenic bacteria and viruses probably interact with influenza. Public health relevance of these pathogen interactions is detailed, showing how possible misreading or a narrow outlook could lead to mistaken public health decision-making. We describe the role of mechanistic transmission models in investigating this complex system and obtaining insight into interactions between influenza and other pathogens. Finally, we highlight the benefits and challenges in modelling and speculate on new opportunities made possible by taking a broader view, including basic science, clinically, and for public health.

## Introduction

Influenza virus is a major contributor to the global disease burden, and exploration of its pathogenesis, epidemiology, and evolution has occupied generations of scientists. Its complex seasonality, antigenic drift of surface proteins, wide spectrum of severity, and capacity to cross species and cause epidemics or pandemics are all characteristics that make the virus so difficult to control [1].

The human respiratory tract is an important reservoir of bacteria, fungi, viruses, bacteriophages, archaea, and eukaryotes [2], harboring diverse communities of commensal, opportunistic, and pathogenic microorganisms. It has been suggested that some exist in nonneutral relationships [3], with competition for resources, synergism with the host immune system, or physiological modifications that alter the normal colonization or infection processes. The contribution of species-level interactions to the influenza burden is largely unknown.

In terms of public health, our current understanding of influenza transmission or severity may therefore be incomplete or misguided due to ignorance of the effect of interacting pathogens. On one hand, large-scale influenza vaccination programs may unexpectedly impact other infections due to an indirect rise or fall in the risk of contracting them [4]. For example, if influenza outcompetes another virus and holds it at bay, an influenza vaccination program could result in an upsurge in the competitor. On the other hand, the introduction of measures to control bacterial infections (e.g., pneumococcal vaccines) may decrease the risk of secondary bacterial pneumonia often associated with severe outcomes of influenza.

Seasonal influenza generates a large burden each year during the wintertime in temperate regions and with more complex seasonal patterns in tropical regions [5]. Influenza pandemics frequently occur outside of the usual season and generate an unpredictable and often large burden in morbidity, mortality, and cost [6,7]. This burden has historically been the result of secondary bacterial infections [8,9]. Lung specimens from 1918 to 1919 influenza fatalities were found to be, in more than 90% of cases, positive for at least one bacterium [10]. Bacteriologic and histopathologic results from published autopsy series also suggest that deaths from the 1918 influenza pandemic mostly resulted from pneumonia with *Streptococcus pneumoniae*, *Haemophilus influenzae*, *Staphylococcus aureus*, and *Streptococcus pyogenes*, multiple infections being common [10]. Deaths during the 1957 and 1968 pandemics were less closely related to bacterial pneumonia [10]. Because emergence and circulation of pandemic influenza take place out of season, and therefore in different climatic and ecological milieus than seasonal strains, pandemic strains may encounter different coinfecting pathogens. It is therefore critically important to pandemic preparedness to understand competitive and synergistic relationships with other species, both at the individual level from a clinical perspective or at a population level from an epidemiological perspective. It is vital to improve our understanding and control of transmission and the risk of developing disease on infection.

Mathematical modelling has been a key tool in infectious diseases for many years, allowing researchers to probe the complex intricacies of transmission and play forward the effects on an individual to see the impact on population-level infection dynamics [11]. Counterfactuals, or ‘what if’ scenarios, can easily be tested and compared, where vaccination rates, contact patterns, health behaviours, or any number of other factors are varied, to assess impact.

Models of influenza virus transmission have proved very useful in expanding knowledge of influenza biology, evolution, and epidemiology. For example, models of evolutionary change and immunity aim to predict the dominant strain of influenza in the coming season [12]. Spatially explicit models have convincingly linked commuting movements to the spread of influenza in the United States [13]. Models have also been crucial to public health, contributing to the optimization of control strategies, including the use of vaccines and antivirals [14–20]. As the modelling field has developed, there has been an effort to improve realism by incorporating heterogeneity in human contact patterns, age-related susceptibility, cross immunity after previous infections [19,21–24], and the potential effect of environmental variables on transmission [13,25]. Notably, the vast majority of modelling work has neglected the microbial environment: most mathematical and computational models of influenza are focused on single or sequential influenza infections and have broadly simplified pathogen ecology. For example, despite secondary bacterial infections being recognized as an important cause of mortality, models have not been exploited to estimate the indirect effect of seasonal influenza vaccination on the incidence of severe bacterial infections in the elderly. Furthermore, modelling used to plan vaccine interventions during the 2009 pandemic in the United Kingdom considered influenza transmission alone [26].

The authors of relatively recent literature reviews gathered biological and epidemiological evidence for interactions between influenza virus and respiratory bacteria or viruses [3,27,28] but did not consider mechanistic transmission models. Mathematical models make it possible to investigate mechanisms of interaction and visualize the pathological and epidemiological patterns that result from them. Comparison of model outputs to data enables estimation of both the probability of such interactions and the strength of the interaction. Estimation can be made across geographic regions (e.g., winter seasonal vs year-round transmission), for different virus subtypes (e.g., seasonal vs pandemic), and in different age groups (e.g., infants vs elderly). Computational and mathematical models to study influenza with other respiratory pathogens are currently underutilized.

In this article, we report evidence of interaction of influenza with other pathogens and systematically review modelling studies on influenza coinfection. Our aim is to build a case for a more expansive use of mathematical models including influenza with other pathogens. For this, we address how different interference mechanisms might lead to unexpected epidemiological patterns and misinterpretations, identify public health needs, identify modelling and biological challenges, and propose avenues of research for the future.

## Mechanisms of interaction

**Fig 1:**
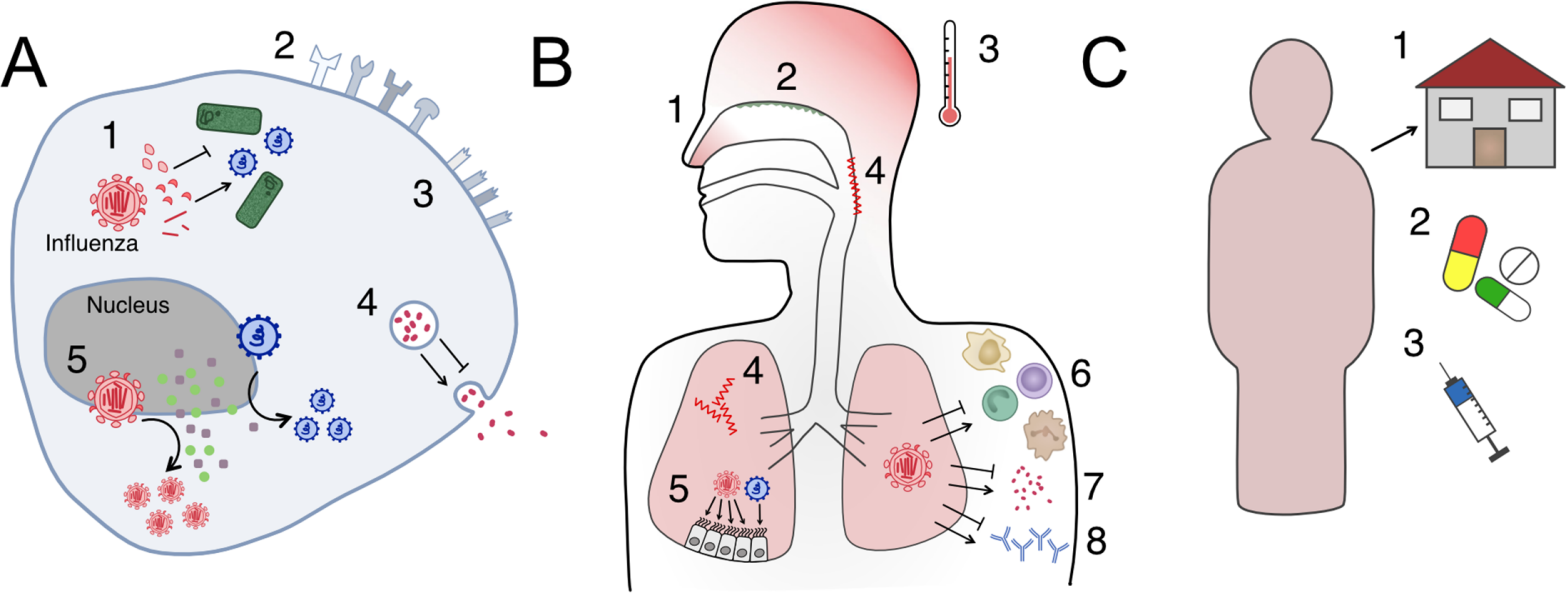
Influenza interactions with other pathogens occur within host or at the population level. Each interaction could either inhibit or enhance coinfection, depending on the combination of pathogens. (A) Cellular-level interactions: (1) direct interactions between viral products; (2) altered receptor presentation; (3) cell damage, e.g., its surface receptors; (4) modification of release of immune system mediators; (5) competition for host resources among influenza and other pathogens. (B) Host-level interactions: (1) change of transmissibility due to symptoms; (2) individual variation in commensal microbiota; (3) effect of symptomatic responses to infection; (4) tissue damage, e.g., in the nasopharynx or lung; (5) competition for host resources, e.g., target cells for infection; (6) immune cell–mediated interaction; (7) immune signalling–mediated interaction; (8) antibody-mediated interaction. (C) Population-level interaction: (1) behavioural responses to disease; (2) medication use; (3) vaccination behaviour. Bacterial interaction mechanisms include A1–5, B1–4 and 7, C1–3. Viral interaction mechanisms include A1–2 and 4–5, B1–3 and 4–8, C1–3.

Here, ‘interaction’ refers to any process by which infection caused by one pathogen affects the probability, timing, or natural history of infection by another. This process includes a wide range of mechanisms that can involve direct connections between the two pathogens, e.g., at the cellular level, or indirect interactions through an intermediate factor that influences the other. The indirect consequences of these interactions are described later. For influenza virus, interactions with bacterial or viral species can occur at several scales (Fig 1). Interacting pathogens may have two distinct profiles: natural human commensals—usually bacteria—which cause mainly asymptomatic carriage or mild symptoms often for long durations of weeks to months, or epidemic pathogens causing infection for shorter durations, from a few days to a few weeks. These two distinct epidemic profiles potentially involve different modes of interaction and lead to different levels of consequences. Here, we detail proven and potential interaction mechanisms (Fig 1).

### Within-host interactions

At the cellular level, interactions involve both direct and indirect mechanisms. First, influenza genes or gene products can enhance or inhibit the replication of other viruses or potential infection by bacteria by direct interaction with pathogen proteins or nucleic acids [29]. Furthermore, indirect competition for host resources can occur, when pathogens compete for target cells, receptors, or cellular products required for replication. Influenza-infected cells may also release cell signalling molecules that could increase or decrease the probability of coinfection.

During infection, influenza virus impairs innate and adaptive host defences [30,31]. Mechanisms include altered neutrophil recruitment and function, leading to defective bacterial clearance, diminished production of alveolar macrophages [32], and inhibition of T cell–mediated immunity [31]. Infection with a second virus could be modulated similarly, e.g., by the production of cross-reactive antibodies or cell-mediated immunity that prevents or facilitates this infection. Physiological changes induced by the host response to infection have consequences on other pathogens. For instance, lung tissue damage [32] and the induction of type-1 interferon signalling were shown to promote bacterial colonization [31] and broadly inhibit viral replication [33]. Damage to lung cells caused by influenza infection, such as influenza neuraminidase stripping sialic acids from the cell surface, amplifies bacterial adherence and invasion [27] and could potentially change the likelihood of infection by another virus. Symptomatic responses to infection, like fever, have also been shown to act as ‘danger signals’ for bacteria, e.g., meningococci, which react by enhancing bacterial defences against human immune cells [34]. In contrast, fever may diminish viral replication rate, thereby lowering the probability of coinfection. From the other side, the ‘influenza preinfection’ respiratory flora of individuals may also partially account for the variability of severity and outcome [28]. For example, *Staphylococcus aureus* colonization was shown to trigger viral load rebounds and reduce influenza virus clearance in animal studies [35–37].

### Population-level interactions

Human behavioural responses to influenza infection can also indirectly impact transmission of bacteria or other viruses. For example, people with severe influenza symptoms are likely to stay home, modifying their contact patterns and making acquisition of second infections unlikely [38,39]. On the other hand, individuals with milder symptoms may maintain their regular activities, which could increase bacterial transmission to other individuals (as observed for tuberculosis [40]) or increase the chance of acquiring a second infection. Person-to-person variation in care seeking and medication use, such as antivirals, antibiotics, antipyretics, or vaccine(s) uptake, also influences the risk of coinfection. For example, use of the pneumococcal conjugate vaccine has decreased carriage of the pneumococcal vaccine strains in some contexts [41,42], and vaccination against *H*. *influenzae* type b has decreased carriage of the bacteria [43,44]. These vaccination campaigns may therefore decrease the chance of observing influenza–bacteria coinfections.

## Evidence of interaction

Several literature reviews have described evidence of interactions between influenza and other respiratory bacterial or viral pathogens [3,27]. In this section, we briefly summarize the viral and bacterial species with evidence for interaction with influenza in recent laboratory and epidemiological studies (details on the search strategies are provided in S1 Appendix, section A).

### Influenza–bacteria interactions

Experimental results suggest that most of the pathogenic and commensal bacteria in the nasopharynx may directly or indirectly interfere with influenza infection during host colonization or infection (Table 1). The best-studied influenza–bacteria interaction is with *Streptococcus pneumoniae* [3]. Influenza is thought to increase bacterial adherence and facilitate the progression from carriage to severe disease [28,45], although evidence from population studies is not so clear-cut [46–49]. Influenza was also shown to impair methicillin-resistant *Staphylococcus aureus* (MRSA) clearance in coinfected mice, thereby increasing their susceptibility to MRSA infection [50]. Similarly, in mice, increased severity of *H*. *influenzae* induced by influenza was suggested, based on experiments of sequential infection with sublethal influenza then *H*. *influenzae* doses [51]. Notably, ecological studies revealed a positive association between influenza and *Neisseria meningitidis* incidence [52] and in vitro studies suggested that direct interaction between influenza A neuraminidase and the *N*. *meningitidis* capsule enhanced bacterial adhesion to cultured epithelial cells [53]. Lastly, in patients with pulmonary tuberculosis, there is evidence of increased risk of severe outcomes on influenza infection [54]. This finding was supported by experiments in mice [55] that demonstrated that *Mycobacterium tuberculosis* and influenza coinfected mice mounted weaker immune responses specific to *M*. *bovis* Bacillus Calmette–Guerin (BCG) in the lungs compared with mice infected with BCG alone.

**Table 1:** Bacteria whose colonization or infection course may be affected by interaction with influenza.

Bacterial species	Study system	Effect	Illustrative publications
*S. pneumoniae*	Animal	Synergistic/Facilitating	Smith 2013 [97]; Wolf 2014 [137]; Siegel 2014 [138]; McCullers 2010 [139]; Ghoneim 2013 [32]; Peltola 2006 [140]; Walters 2016 [141]; Nakamura 2011 [142];
	Human	Synergistic/Facilitating	Walter 2010 [143]; Nelson 2012 [144]; Opatowski 2013 [102]; Shrestha 2013 [107]; Weinberger 2013 [89]; Jansen 2008 [145]; Kuster 2011 [146]; Nicoli 2013 [88]; Ampofo 2008 [147]; Grabowska 2006 [148]; Murdoch 2008 [149]; Edwards 2011 [150]; Weinberger 2014 [151]; Grijalva 2014 [152];
		Neutral/Unclear	Kim et al. 1996 [47]; Watson 2006 [48]; Toschke 2008 [49]; Zhou 2012 [153]; Damasio 2015 [154]; Hendricks 2017 [91]
*S. aureus*	In vitro and Animal	Synergistic/Facilitating	Niemann 2012 [155]; Davison 1982 [156]; Tashiro 1987 [37]; Zhang 1996 [157]; Chertow 2016 [158]; Sun 2014 [50]; Braun 2007 [35]; Iverson 2011 [159]; Robinson 2013 [160]
	Human	Synergistic/Facilitating	Sherertz 1996 [161]; Hageman 2006 [162]; Finelli 2008 [163]; Reed 2009 [164]
		Neutral	Kobayashi 2013 [165]
*H. influenzae*	Animal	Synergistic/Facilitating	Lee 2010 [51]; Michaels 1977 [166]; Bakaletz 1988 [167]; Francis 1945 [168]
	Human	Synergistic/Facilitating	Morens 2008 [10]
*N. meningitidis*	In vitro and Animal	Synergistic/Facilitating	Rameix-Welti 2009 [53]; Loh 2013 [34]
		Neutral	Read 1999 [169]
	Human	Synergistic/Facilitating	Cartwright 1991 [170]; Hubert 1992 [52]; Jacobs 2014 [171]; Brundage 2006 [172]; Jansen 2008 [145]; Jacobs 2014 [171]; Makras 2001 [173]
*M. tuberculosis*	Animal	Synergistic/Facilitating	Florido 2015 [174]; Florido 2013[55]; Volkert 1947 [175]; Redford 2014 [176]
	Human	Synergistic/Facilitating	Walaza 2015 [177]; Oei 2012[178]; Noymer 2011 [179]; Noymer 2009 [180]; Zurcher 2016 [181]
		Neutral	Roth 2013[182]
*S. pyogenes*	Animal	Synergistic/Facilitating	Klonoski 2014 [183]; Okamoto 2003 [184]; Okamoto 2004 [185]; Hafez 2010 [186]
	Human	Synergistic/Facilitating	Scaber 2011 [187]; Zakikhany 2011 [111]; Tasher 2011 [188]
		Neutral	Tamayo 2016 [189]

### Virus–virus interactions

Within its family, influenza interacts between types (A and B), subtypes (e.g., H3N2, H1N1), and strains. Competitive exclusion due to homologous immunity is widely accepted [56,57] and has been applied extensively in models of influenza strain coexistence [58,59]. Antigenic change (measured through antigenic distance) occurs constantly in influenza, strongly indicating that the virus escapes from immunity resulting from prior infection by genetic change [60]. Interestingly, there is mounting evidence that the first influenza infection is important and may affect severity of future infections [61–63]. Some evidence also supports the finding that influenza can interact with other influenza viruses and noninfluenza respiratory viruses via nonspecific immunity following infection [64,65].

Many noninfluenza viruses are also suspected of interfering with influenza virus acquisition, based on different types of studies (Table 2). During the 2009 influenza pandemic, Casalegno et al. reported that in France, the second pandemic wave was delayed due to the September rhinovirus epidemic [66], although this shift was not observed in other countries [67,68] and may have been affected by variable reporting rates. Coinfection by the two viruses might also enhance disease severity for individuals [69–71], although evidence is discordant [72–74]. Similarly, competitive interaction with respiratory syncytial virus (RSV) has been posited for many years [75,76], and some evidence was found for delayed RSV epidemics due to the second wave of the 2009 pandemic in France [77] and tropical regions [78,79]. There is discrepancy in the findings of interaction between influenza and RSV; while most studies found increased severity [74,80,81], others found no effect [69] and some found less severity [82]. Competitive interaction with parainfluenza viruses was also inferred, based on less frequent coinfection pairs than expected [83], but that observation is not consistent across studies [84–86]. In terms of severity, parainfluenza and influenza coinfection is usually more severe than influenza alone [69,71,87] but not always [72,73].

**Table 2:** Viruses that may be affected by interaction with influenza.

Virus	Study system	Effect	Illustrative publications
RSV	Population incidence	Competitive	Anestad 2007 [190]; Anestad 2009 [191]; Casalegno 2010 [77];, Anestad 1987 [192]; Yang 2012 [79]; Nishimura 2005 [193]; Glezen 1980 [76]; Pascalis 2012 [83]; Yang 2015 [67]; van Asten 2016 [194]; Meningher 2014 [195]; Velasco-Hernandez 2015 [117]
		Neutral	Navarro-Mari 2012 [68]
	Coinfection detection	Competitive	Greer 2009 [84]; Martin 2013 [196]
	Laboratory investigation	Competitive	Shinjoh 2000 [197]
Rhinovirus	Population incidence	Competitive	Casalegno 2010 [66]; Casalegno 2010 [77]; Pascalis 2012 [83]; Linde 2009 [198]; Anestad and Nordbo [199]; Cowling 2012 [65]; Yang 2015 [67]
		Neutral	Yang 2012 [79]; Navarro-Mari 2012 [68]; van Asten 2016 [194]
	Coinfection detection	Competitive	Tanner 2012 [200]; Mackay 2013 [201]; Nisi 2010 [86]; Greer 2009 [84]; Martin 2013 [196]
	Laboratory investigation	Competitive	Pinky and Dobrovolny 2016 [112]
Influenza	Population incidence	Competitive	van Asten 2016 [194]
	Coinfection detection	Competitive	Nisii 2010 [86]; Sonoguchi 1985 [56]
	Laboratory studies	Competitive	Easton 2011 [202]; Laurie 2015 [57]
HPIV	Population incidence	Competitive	Yang 2012 [67]; Anestad 1987 [192]; Yang 2015 [67]
		Neutral	Mak 2012 [78]
	Coinfection detection	Competitive	Pascalis 2012 [83]
		Neutral	Murphy 1975 [85]; Nisii 2010 [86]; Greer 2009 [84]; Martin 2013 [196]
	Laboratory investigation	Synergistic/Facilitating	Goto 2016 [203]

Abbreviations: HPIV, human parainfluenza virus; RSV, respiratory syncytial virus.

The general pattern is that bacteria tend to synergize with influenza, often boosting transmission of either pathogen or increasing invasion of the bacteria following influenza infection. It is not always clear whether this is a true synergy—in which both pathogens benefit—or rather that influenza facilitates bacterial invasion. In contrast, viral pathogens tend to form competitive interactions with influenza, although whether these are direct, specific interactions with particular other viruses or the result of an ‘early advantage’ to the first infector remains unclear. This pattern may occur because of the differing natural histories of bacteria and viruses; while the former tends to infect hosts for long time periods, the latter has shorter infections more similar to the natural history of influenza itself. This is a complex system in which each host–pathogen or pathogen–pathogen interaction phenomenon may impact the others. Surprisingly, however, such interactions remain poorly studied and, in particular, very few modelling studies have addressed these questions.

## Impact of interactions at the population level

**Fig 2:**
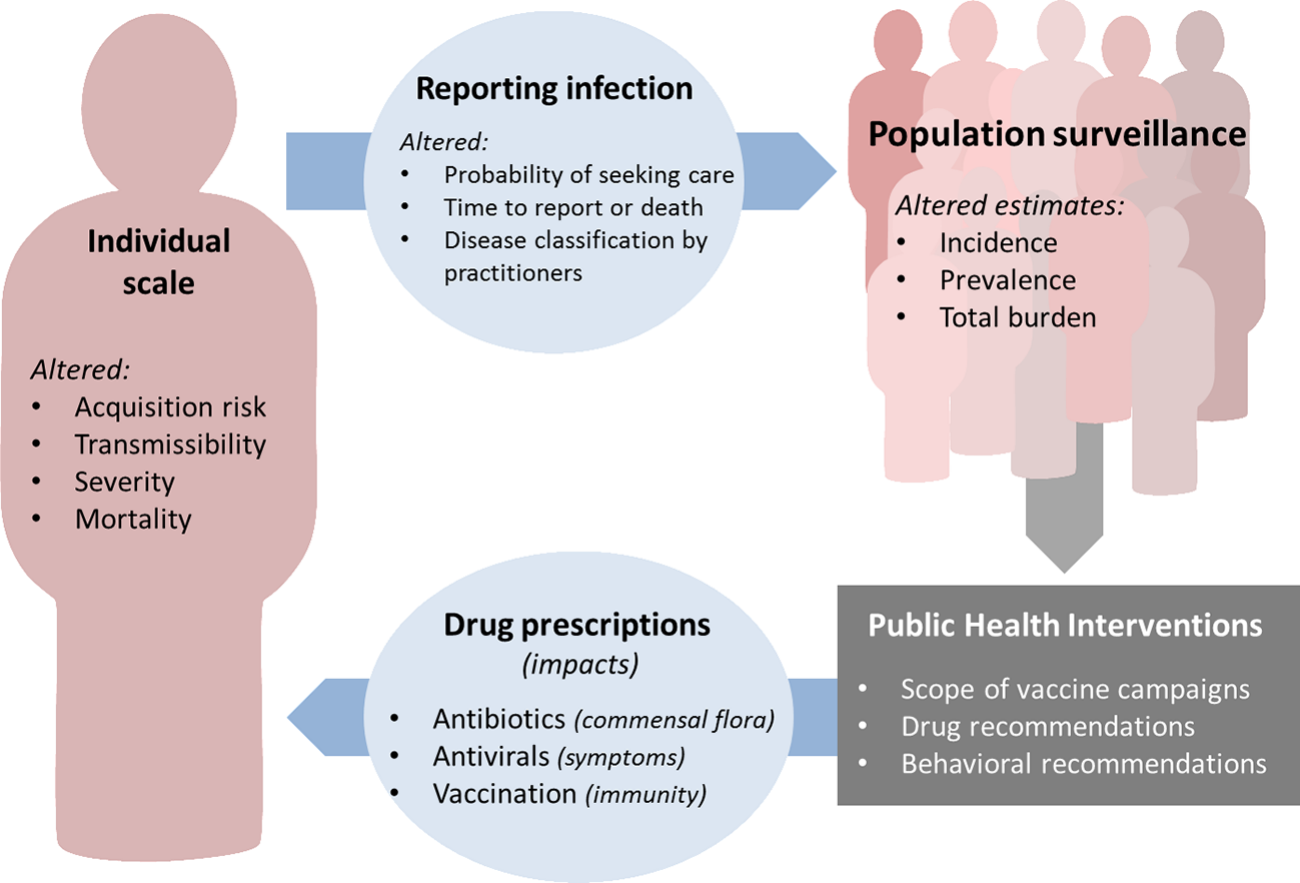
Cycle of factors affected by nonneutral interactions at the individual level and their impact on influenza surveillance, treatment, prevention, and control. Factors that affect coinfection on an individual scale can feed forward to an effect on population surveillance through their effects on the reporting of infection. Decisions on public health interventions are made in response to population-level data. These interventions then take effect at the individual level, to give a feedback loop both generated and impacted by effects of coinfection.

Although coinfections occur at the host level, their consequences are far-reaching (Fig 2). Coinfection may alter the natural history, severity, or timing of illness in an individual and thereby modify the morbidity, healthcare-seeking behaviour, and treatment of that individual. Heterogeneity in these can affect the probability of, and timing of, reporting disease, thereby transferring the effect from individual hosts to the population level.

Development and implementation of public health policies rely on analyses of population surveillance data on influenza epidemics and burden. Policies then generate changes in medical interventions at the population level, e.g., change in vaccination targets, or at the individual level, e.g., recommendations for antibiotics or antivirals in certain groups. These public health interventions then have their own impacts on the dynamics of pathogens and coinfections. Therefore, because coinfections may alter surveillance data, and policies based on evidence from surveillance data may alter coinfection or interference risk, there is a complex cycle of dependence, which highlights the difficulty—as well as the potential importance—of assessing the impact of coinfections (Fig 2).

To date, most of the published quantitative analyses of interactions rely on statistical association between incident cases of influenza-like illness (ILI) and other infections based on regression and correlation analyses [88,89]. A major methodological challenge of detecting interactions is that significant correlation between epidemics of two pathogens in surveillance data may result from either a true biological direct or indirect interaction or may be confounding as a result of the two pathogens sharing common ecological conditions (e.g., cold weather). Regression models describe simple functional links between, for example, the incidence time series, onset or peak time, or epidemic magnitude or severity. Despite their apparently simple formulation, they rely on strong statistical assumptions on the shape of the data and the association [90]. Regression models are also used to calculate correlations between reported time series at different time lags. When properly controlled for confounding variables, they have proved very useful tools to detect signals of associations. Other methods have been proposed through the deployment of seasonal autoregressive integrated moving average (SARIMA) models to analyse time series [91], Granger causality [92,93], or seasonality patterns [94]. However, these models do not formalize the transmission process or biological mechanism of interaction, so the interaction mechanism cannot be determined nor the strength of interaction quantified. Furthermore, this lack of mechanistic formulation prevents easily interpretable predictions that are required to support public health decision-making.

Due to the complex phenomena and many feedback loops, mechanistic models are needed to dissect the cause and effect of the different components (Box 1) [95]. The role of modelling is two-fold: first, mathematical modelling provides a common language to integrate heterogeneous mechanisms and test competitive hypotheses. By doing so, models contribute to building basic knowledge about infection processes. Second, modelling enables assessment of potential intervention scenarios by predicting their impact.

**Box 1. Mathematical modelling definitions**

**Mathematical** versus **statistical models**: A **mathematical model** (or **transmission** or **mechanistic model**) is a mechanistic description by mathematical equations of how the number of infected entities changes over time. For example, a mathematical model of transmission between people might explicitly track the number of infected people and describe how many contacts they make, how often these contacts lead to transmission, and how this is affected by temperature. Depending on the scale of the model, entities can be cells, individuals, or groups of individuals (e.g., a household, a city). **Statistical models** do not include a mechanistic link between quantities but only rely on an observed association, often in the form of a probability distribution. So, in the case of the statistical model, you might say that you see more cases when the temperature is low, without explicitly explaining why.

**Individual-based model** versus **compartmental models**: **Individual-based models** (or **agent-based models**) include a description of the properties (e.g., age, immune status, risk factors) of each of the individuals in the studied population. In contrast, **compartmental models** group individuals with similar characteristics together into compartments and look at relationships between these compartments. The most famous compartmental in epidemiology is the SIR model, based on three compartments, Susceptible-Infectious-Recovered, which is the basis of most of the existing models of pathogen transmission. Compartmental models are easier to fit to data (see next section) and interpret. Individual-based models are more flexible when it is important to integrate a wide range of characteristics of the population but are comparatively slow to implement and run, more difficult to interpret, and require good data on each characteristic that is modelled.

**Model fitting**: Models are built around a structure (the mechanisms), which is modulated by parameters governing the rates of change between compartments, disease states, behaviours, etc. Historically, parameters have been estimated using results from studies published in the literature. In recent years, with the increased availability of epidemiological data, modelers try whenever possible to fit the model to data (also called **parameter inference** or **calibration**). For this, they use algorithms that explore ‘parameter space’, which is the set of all possible values for parameters, and retain sets of parameters that explain the observed data best. Fitting can be computationally intensive if the model includes many parameters. More efficient fitting algorithms allow fitting of more complex models and thus the study of potentially more interaction mechanisms.

For these reasons, public health interventions based on modelling of infectious diseases have become informative and effective. For example, in the UK, a transmission model fitted to a vast range of ILI and influenza surveillance data demonstrated that vaccinating children against influenza will have the same protective effect on people over 65 years old as vaccinating those individuals [96]. This outcome is a consequence of the diminished community transmission that results from reducing infections in children. Such an impact would be impossible to identify without mechanistic models. Box 2 summarises the potential benefits of coinfection transmission models.

**Box 2. Benefits of coinfection transmission models**

- Allow causal relationships to be drawn from the data by testing hypotheses regarding interaction mechanisms
  ○ For example, using models to analyse the cellular dynamics observed in vivo in mouse coinfection experiments, it is possible to design models of hypothesised immunological pathways and determine which most closely fits observed patterns [97].
- Evaluate contributions to influenza burden with more precision
  ○ For example, year-to-year influenza epidemics have a different estimated reporting fraction. A model could be used to determine whether coinfection or concurrent epidemics of other viruses are the reason for an increased (or decreased) probability of reporting infection.
- Predict or project incidence of coinfections, including during pandemics
  ○ For example, fitting multipathogen models to respiratory virus surveillance data would allow quantitative assessment of the hypothesis that during the 2009 pandemic, influenza affected the timing of rhinovirus, RSV, and influenza by competition [66,77].
- Optimize prevention and control of influenza infections and their complications
  ○ For example, a model of influenza and pneumococcal pneumonia could determine optimal target groups for pneumococcal vaccination, based on both the bacterial carriage rates in each age group and the expected influenza vaccination rates in those age groups.
- Estimate the costs and benefits of intervention strategies
  ○ For example, a model-based analysis of in vitro experimental data could allow assessment of the impact of early antiviral or antibiotic treatment on probability of pneumococcal invasion [98,100]. Combined with population, it would be possible to assess the impact on secondary bacterial infections.

## Models of influenza interactions

Despite mounting evidence of influenza–bacteria interactions and the concurrent increasing use of dynamic modelling to study infectious diseases in recent decades, influenza interactions have rarely been modelled. Interestingly, previous literature reviews describing evidence of interactions between influenza virus and other respiratory bacterial or viral pathogens neglected mathematical models that, despite their limited number, provide insight into mechanisms of interaction and their consequences [3,27]. We have systematically reviewed the literature for models incorporating influenza with bacteria or noninfluenza viruses (details on the search strategies are provided in S1 Appendix, section A).

### Influenza–bacteria interaction

The only influenza–bacterium interaction that has been integrated into mathematical modelling studies is the influenza–pneumococcus system, both within host and at the population level.

Several dynamic models of coinfection at the cellular level were proposed relatively recently [97–101]. In a study combining modelling and empirical data from mice coinfected with two different influenza viruses and two pneumococcus strains, Smith et al. assessed the likelihood of different immunological interaction mechanisms [97]. They found a role of macrophage dysfunction leading to an increase of bacterial titres and increased virus release during coinfections [97], although their results suggest that coinfection-induced increase of bacterial adherence and of infected cell death were not very likely. Shrestha et al. used an immune-mediated model of the virus–bacterium interaction in the lungs to specifically quantify interaction timing and intensity [98]. They assumed that the efficiency of alveolar macrophages, which are a critical component of host immunity against bacterial infections, was reduced by viral infection and tested the impact of inoculum size, time of bacterial invasion after influenza infection, and the potential impact of antiviral administration. The model predicted that enhanced susceptibility to invasion would be observed four to six days after influenza infection, suggesting that early antiviral administration after influenza infection (<4 days) could prevent invasive pneumococcal disease. Smith and Smith modelled a nonlinear initial dose threshold, below which bacteria (pneumococcus) declined and above which bacteria increased. Using data from mice experiments, they showed that this threshold was dependent on the degree of virus-induced depletion of alveolar macrophages. Because macrophage depletion varies through the course of influenza infection, this important finding may explain why risk of bacterial invasion also changes over the course of infection, with particularly low dose requirement in the first few days of infection [99]. In a follow-up study, the same authors analysed published data from influenza–pneumococcus coinfected mice treated with antiviral, antibiotic, or immune modulatory agents. They found that antivirals are more efficient at preventing secondary infection when used in the first two days of influenza infection and also found an important benefit of immunotherapy, especially for low bacterial loads [100]. Lastly, in a within-host model, Boianelli and colleagues investigated the efficacy of different oseltamivir treatment regimens in influenza–pneumococcus coinfected individuals using parameters drawn from human and mouse studies. They found that increasing the dose of oseltamivir, but not duration of treatment, might increase both its antiviral and antibacterial efficacy [101].

At the population level, there have been several models to assess influenza interactions with bacteria and test hypotheses regarding the main mechanisms [102–106]. The comparison of pneumococcal transmission models to analyse time series of pneumococcal meningitis and viral respiratory infections in France highlighted two important processes in colonized individuals: (1) a virus-related increase in pneumococcal pathogenicity and (2) an enhanced between-individual transmissibility of bacteria [102]. Models of transmission of bacterial pneumonia fitted to US data also highlighted significant interactions, mainly due to influenza-associated increase of individual risk of pneumonia [103,107]. Recently, in a simulation study, Arduin et al. used a flexible individual-based model of influenza–bacteria interaction to assess the population consequences and associated burden of a range of pneumococcus–influenza interaction mechanisms [108]. Population dynamic models have also been used to test the public health impact of control measures [104–106]. Different strategies of antibiotic use (as treatment or prophylaxis) and of vaccination were assessed by modelling the dual transmission of pneumococcus and influenza [104]. For a 1918-like pandemic, this model suggested that widespread antibiotic treatment of individuals with pneumonia would significantly lower mortality, whereas antibiotics in prophylaxis would effectively prevent pneumonia cases. A different model evaluated the benefit of vaccinating the UK population against pneumococcus in the context of pandemic influenza using different scenarios: 1918-like, 1957/1968-like, or 2009-like virus [105]. This indicated that pneumococcal vaccination would have a major impact only for a pandemic with high case fatality and secondary pneumococcal infection rates (e.g., the 1918-like), with less influence in other scenarios.

### Viral interaction

Influenza–influenza interactions predominate in models of two viruses, with limited investigation of influenza–RSV interactions and no models of other viruses.

Within host, several models of multistrain influenza infections were proposed [109–111], especially examining the interval before the secondary infection. One model of RSV–influenza interaction at the cellular level explored the hypothesis of the viruses interacting through competition for resources within the cell [112]. This indirect competition was sufficient to explain the observed rate of virus replication. The model also explored how the speed of virus replication confers an advantage to the first infecting pathogen and determined the ‘head start’ on infection that the slower-replicating virus would require to maintain dominance.

Population models have been used extensively to examine the dynamics of influenza and multistrain influenza systems (for a review see [113]) although many fewer studies examined multispecies systems. Because the influenza virus comprises two types, multiple subtypes, and potentially numerous strains of each, many viruses may be circulating at any given time, providing varying degrees of cross-protection after recovery and sometimes with complex dynamics of within-species strain replacement due to genetic drift or reassortment. There is evidence of competition between strains, with some models requiring short periods of heterologous immunity after infection to create the ladder-like phylodynamic structure of influenza viruses [114], although recent studies could capture this feature without this mechanism [58]. One comprehensive early model tested four mechanisms of interaction between influenza types using data from Tecumseh, Michigan, but the data were insufficient to distinguish the mechanisms [115]. Influenza–influenza models must also account for the complex immune history of hosts, related to which there is mounting evidence that the timing of an individual’s influenza encounters, and especially the first infection, shapes their future response [61–63]. The methods for modelling influenza–influenza interactions should be extended into interactions with other viruses.

One model for pandemic influenza, in which coinfection with other respiratory pathogens leads to enhanced influenza transmission, was proposed to explain the multiple waves of the 1918 influenza pandemic in the UK [116]. A recent example of influenza and RSV cross-species analysis in a climatically driven model provided some evidence that RSV dominates influenza, but the model was not explicitly fitted to data [117].

### Illustration from a simple model

**Fig 3:**
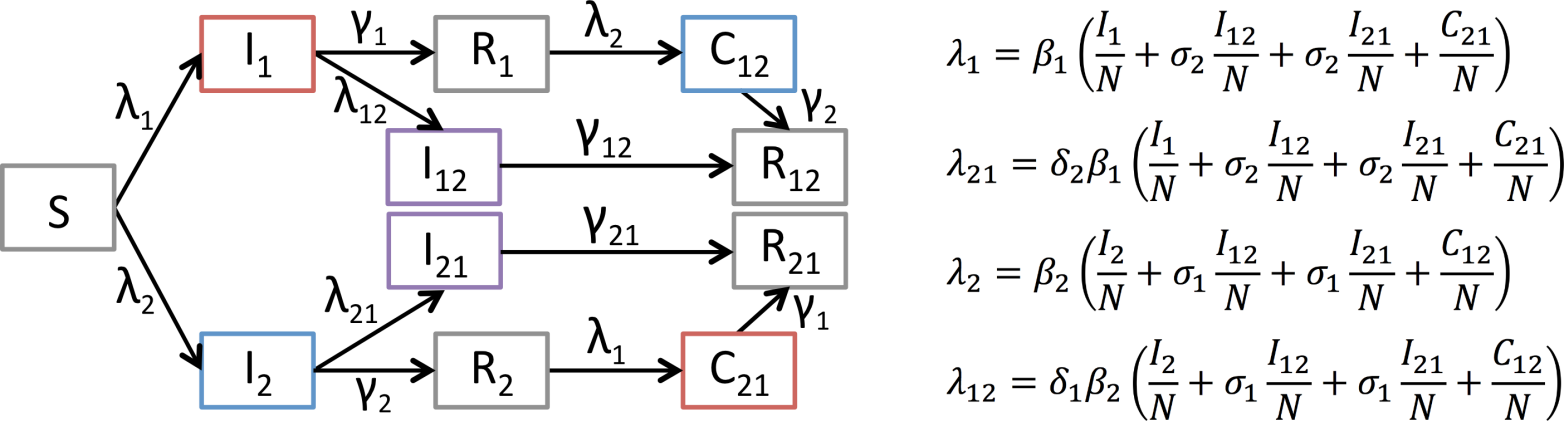
Illustration of a simple model of two circulating pathogens in interactions. Schematic of the compartments and rates of transition between compartments, with equations of the forces of infection by pathogen 1 (λ_1_), pathogen 2 (λ_2_) for susceptible hosts, and pathogen 1 (λ_21_) and pathogen 2 (λ_12_) for hosts already infected by the other pathogen. The full system of ordinary differential equations describing the changes of the compartment’s populations over time is described in S1 Appendix, section B. Details of the model and parameters are provided in Box 3.

**Fig 4:**
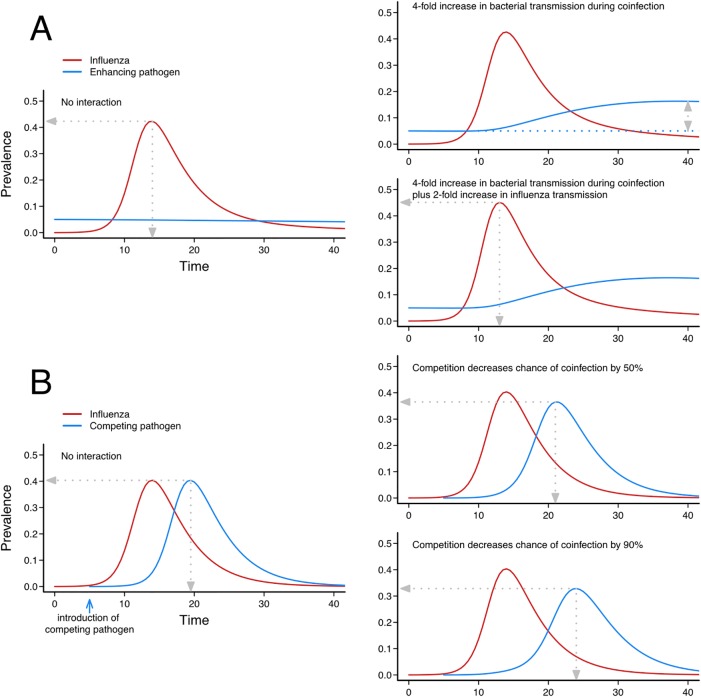
Example model outputs showing effect of synergistic and competitive interaction. Box 3 gives details on the model that produces these epidemic trajectories. (A) In the baseline enhancing scenario, an endemic bacterial pathogen (blue) occurs at 5% prevalence. An influenza epidemic occurs with no interaction, and the bacterial prevalence does not change. If the presence of influenza coinfection increases bacterial transmissibility by 4-fold (*σ*_*1*_ = 4), then there is a transient rise in bacterial prevalence. If there is also an increase in influenza transmissibility during coinfection (*σ*_*1*_ = 4 and *σ*_*2*_ = 2), then there is also a higher and earlier influenza peak as a result of coinfection. (B) In the baseline competition scenario, the second epidemic pathogen is introduced later than influenza. The two pathogens have the same transmission characteristics (same *γ*, same β). If there is only a 50% chance of infection with pathogen 2 when individuals are infected with pathogen 1 (*δ*_*1*_ = 0.5), then the epidemic trajectory of pathogen 2 is lower and later. If competition is even stronger (*δ*_*1*_ = 0.1) so there is a 90% reduction in chance of coinfection, the profile of pathogen 2 is even further separated from pathogen 1. Computer code generating these trajectories is given in S1 Code.

To demonstrate how both synergistic and competitive interactions can be modelled, we used a simple transmission model and simulated the effect of interactions (Box 3, Figs 3 and 4 and S1 Appendix, section B). We show how these interactions occurring at the individual level can impact the epidemics at the population level. The ‘bacterial type’ interaction firstly shows an increase in bacterial prevalence when influenza infection increases bacterial transmission, in a facilitative interaction. In a synergistic interaction, where coinfection increases transmission of both influenza and bacteria, prevalence of bacteria increases, and the epidemic of influenza has a quicker and higher peak. In the ‘viral type’ competitive interaction, progressively decreasing the probability that a second pathogen can infect an already infected host causes the epidemic peaks to separate in time. It also decreases the peak size of the outcompeted pathogen without altering the number of people infected in total (Fig 4).

**Box 3. A simple model of interaction**

The simple model in Fig 3 tests two interaction mechanisms: increased (or decreased) infectiousness on coinfection and decreased (or increased) probability of coinfection occurring. These are the two most commonly suggested mechanisms, the first of the ‘bacterial type’ and the second of the ‘viral type’ (Fig 4).

In Fig 3, all individuals start in the Susceptible (S) class and move to the Infectious classes when they are infected by either pathogen 1 (*I*_*1*_) or 2 (*I*_*2*_).

Infected (and infectious) compartments are shown in colour, where red is infectious with pathogen 1, blue marks infectious with pathogen 2, and infected and infectious with both pathogens in purple. Infection rates are given by the four forces of infection (*λ*_*1*_, *λ*_*2*_, *λ*_*12*_, *λ*_*21*_). After being infected by one pathogen, individuals can either be coinfected by the other pathogen and move to the coinfection compartments in purple (*I*_*12*_ or *I*_*21*_), or they can recover at rates *γ* and move to the Recovered compartments (*R*_*1*_ and *R*_*2*_). Coinfected individuals (*I*_*12*_ and *I*_*21*_) recover and remain in the doubly recovered compartments, *R*_*12*_ and *R*_*21*_. Individuals in *R*_*1*_ or *R*_*2*_ are subject to force of infection *λ*_*2*_ or *λ*_*1*_, respectively, i.e., of the pathogen they have not yet had. On infection with the other pathogen, they move to the consecutive infection compartment (*C*_*12*_ or *C*_*21*_). After recovery, those individuals move to the doubly recovered compartments (*R*_*12*_ and *R*_*21*_).

Parameters *β*_*1*_ and *β*_*2*_ are the baseline transmissibility of pathogen 1 and 2, respectively. There are four interaction parameters modulating the pathogen’s transmissibility: *σ*_*1*_ and *σ*_*2*_ are the change in infectiousness of coinfected classes, where a value less than 1 makes the coinfected class less infectious, and a value greater than 1 means coinfected individuals are more infectious. Parameters *δ*_*1*_ and *δ*_*2*_ alter the probability of acquisition of a second infection following a first infection, where a value less than 1 makes coinfection less likely, and a value above 1 makes it more likely.

Details on the model equations and computer code generating the trajectories are given in S1 Appendix, section B and S1 Code.

## Limits of the current view

Historically, scientific and medical studies have tended to focus on host–pathogen interactions in an independent manner by studying each pathogen alone. We highlighted here, as others [3,27,28], that many respiratory viruses and bacteria have been linked to influenza epidemiology, based on in vivo evidence and from individual and epidemiological studies. These nonneutral interactions, mostly facilitative for bacteria and competitive for viruses, probably have individual- and population-level effects on influenza pathogenicity, burden, and potentially its epidemic profile.

Mathematical models are crucial to guide public health decision makers, who, for ethical or cost reasons, cannot conduct large-scale trials. Two examples of interventions based on modelling results and mobilizing important public resources are pandemic preparedness (stockpiling of antivirals, use of vaccine doses) [118] and national immunization programs [20]. Neglecting the cocirculating pathogens—i.e., adopting influenza tunnel vision—and the indirect impact of coinfections may potentially affect the estimation of the risk associated with influenza infection and, consequently, the accuracy of model predictions. Interaction strength may also change from year to year and depend on the circulating influenza strains. For evaluation of interventions, this neglect can lead to overestimation of the impact—if burden was measured without considering the changing landscape of coinfection in the population—or underestimation—if the effect of an intervention does not account for the potentially decreased burden of an interacting pathogen as a result of diminished influenza transmission. For all these reasons, we think that adopting a more holistic approach to modelling of respiratory pathogens will improve their surveillance and the strategy to control them.

## Opportunities

Considering influenza virus in its ecological context and its interactions as a cause of the associated morbidity and mortality should offer opportunities for prevention and treatment. In addition to influenza vaccines that (partially) protect against infection, antibacterial vaccines are also critical. For example, pneumococcal vaccines have been shown to have good efficacy against influenza-associated nonbacteremic pneumonias [119,120]. The 23-valent pneumococcal polysaccharide vaccine significantly lowered the risk of invasive pneumococcal disease and attributed mortality in the elderly [121]. Better understanding of possible influenza–pneumococcus interactions and integrating those into transmission models could potentially enable us to identify synergies between vaccination programs and optimize the use of both vaccines.

In addition, there may be opportunities for optimization of antibiotic and antiviral prescriptions. First, antibiotics have historically been used to prevent secondary infections [122,123]. Increasing rates of antibiotic resistance worldwide led to policies to decrease antibiotic consumption, focusing particular attention on reducing prescriptions for viral infections. Second, neuraminidase inhibitors were found to prevent some secondary bacterial pneumonias in animal experiments, human epidemiological studies, and mathematical modelling studies, beyond the window in which they directly impact the influenza viral load [98,124,125]. Although antivirals may only modestly attenuate influenza symptoms, a body of evidence suggests they could avoid severe and economically important outcomes of influenza infection [125–128].

Lastly, accurate burden quantification is crucial to designing and implementing public health interventions against influenza. Focusing efforts to better understand these interactions is therefore critical, especially in the context of pandemic influenza but also to plan for seasonal epidemics, by forecasting the onset and peak times and estimating the expected burden. To improve our knowledge, models can be used to analyse available surveillance and experimental data, generate hypotheses regarding interaction mechanisms at play in transmission or infection, and test their likelihoods. Competing assumptions on the biological interaction processes can be assessed, and the strength of interactions can also be estimated. From a public health viewpoint, such models would help better estimate the burden of influenza virus interactions in terms of morbidity and mortality, the cost-effectiveness of interventions, and, critically, more accurately predict the real impact of control measures.

## Challenges

Integrating transmission and infection by multiple pathogens into mathematical models poses several challenges. From a methodological perspective, modelling several pathogens with interrelated natural histories makes classical compartmental approaches more difficult. Individual-based frameworks (Box 1) are better adapted for this task. For example, this approach could be used to investigate the effect of the interval between influenza infection and bacterial acquisition, which reportedly affects the risk of bacterial invasion [31,129]. Individual-based models are often more computationally intensive and can introduce new difficulties in terms of parameter estimation, requiring the design of new methods. Recent developments in statistical inference methods, like particle Markov chain Monte Carlo (pMCMC) or maximum likelihood estimation via iterated filtering (MIF) [130,131], now enable modelers to jointly fit complex population-based models to multiple types of data, thereby allowing more data and more diverse types of data to inform the model parameters.

Epidemiological data represent the second major challenge. To date, modelling studies have been limited by the poor knowledge of respiratory viruses and bacteria circulating in the community, especially because little is known about prevalence, incidence, at-risk populations, and even epidemic profiles in different populations. Deeper understanding of the ecology of the vast number of microorganisms that can contribute is needed. On an individual level, new studies are required to assess the effect of coinfections rather than ecological associations from incidence data. Important features include (i) coinfection-induced alteration of diseases’ natural histories, e.g., increased acquisition and severity risk, changes of infection durations and generation times; (ii) specific at-risk periods for secondary infection or invasion of the coinfecting pathogen, or at-risk periods for severe outcomes; and (iii) at-risk populations, as characterized by individuals’ age, comorbidities, or behavioural risk factors.

For population-level data, in most countries, surveillance of influenza acquisition is based on networks of general practitioners who notify patients consulting for clinical symptoms of ILI [132]. Surveillance data streams based on syndromic surveillance [133], inpatient data [134], and pathogen testing [135,136] should be combined, and linked at the patient level, to better identify noninfluenza infections or anomalous epidemics that could signal interaction. Improvement of data quality in patient records and detection of the biases inherent in different types of surveillance data are critical to achieve this goal. The latter could be reached by developing new microbiological tools, including new sampling kits able to rapidly detect multiple pathogens for use during medical consultations.

Public health decision-making for interacting pathogens is a difficult but important challenge. When multiple competing treatment options are available, a coherent framework is needed to determine the best strategy. While the question goes beyond influenza to interactions and coinfections for respiratory viruses in general (e.g., RSV), influenza is one of the most studied viral infections and is therefore the ideal first candidate to develop a more holistic mathematical modelling approach.

## Conclusion

In this study, we examined the epidemiological and biological evidence supporting influenza virus interference and interaction with other pathogens. We highlighted opportunities to improve knowledge and control of the virus, if we can move forward from the tunnel vision of single-pathogen models. It is time to develop a more holistic approach to pathogen dynamics in mathematical modelling, with novel methodological innovations, and further efforts in data collection and surveillance. The motivation to do so lies in the real opportunity to improve public health practices and create better, more cost-effective interventions against influenza.

## Supporting information

S1 Fig PRISMA diagram for search for influenza–bacteria interactions. (TIF)

S2 Fig PRISMA diagram for search for influenza–other virus interactions. (TIF)

S1 Appendix Microsoft Word document providing details on the search strategy and the mathematical model. (DOCX)

S1 Code Computer code of the coinfection model. (R)

## References

[1] Webster RG, Monto A. S., Braciale T. J. & Lamb R. A. (2013) Textbook of Influenza.

[2] de Steenhuijsen Piters WAA, Sanders EAM, Bogaert D, Grice E, Segre J, et al (2015) The role of the local microbial ecosystem in respiratory health and disease. Philosophical transactions of the Royal Society of London Series B, Biological sciences 370: 244–253. 10.1098/rstb.2014.0294 PMC4528492 26150660

[3] Bosch AATM, Biesbroek G, Trzcinski K, Sanders EAM, Bogaert D (2013) Viral and bacterial interactions in the upper respiratory tract. PLoS Pathog 9: e1003057 doi: 10.1371/journal.ppat.1003057 23326226 10.1371/journal.ppat.1003057 PMC3542149

[4] Yamin D, Balicer RD, Galvani AP (2014) Cost-effectiveness of influenza vaccination in prior pneumonia patients in Israel. Vaccine 32: 4198–4205. doi: 10.1016/j.vaccine.2014.05.015 24930716 10.1016/j.vaccine.2014.05.015 PMC4077912

[5] Bedford T, Riley S, Barr IG, Broor S, Chadha M, et al (2015) Global circulation patterns of seasonal influenza viruses vary with antigenic drift. Nature 523: 217–220. doi: 10.1038/nature14460 26053121 10.1038/nature14460 PMC4499780

[6] Hayward AC, Fragaszy EB, Bermingham A, Wang L, Copas A, et al (2014) Comparative community burden and severity of seasonal and pandemic influenza: results of the Flu Watch cohort study. Lancet Respir Med 2: 445–454. doi: 10.1016/S2213-2600(14)70034-7 24717637 10.1016/S2213-2600(14)70034-7 PMC7164821

[7] de Francisco Shapovalova N, Donadel M, Jit M, Hutubessy R (2015) A systematic review of the social and economic burden of influenza in low- and middle-income countries. Vaccine 33: 6537–6544. doi: 10.1016/j.vaccine.2015.10.066 26597032 10.1016/j.vaccine.2015.10.066

[8] Brundage JF, Shanks GD (2008) Deaths from bacterial pneumonia during 1918–19 influenza pandemic. Emerging Infectious Diseases 14: 1193–1199. doi: 10.3201/eid1408.071313 18680641 10.3201/eid1408.071313 PMC2600384

[9] Joseph C, Togawa Y, Shindo N (2013) Bacterial and viral infections associated with influenza. Influenza and Other Respiratory Viruses 7: 105–113. doi: 10.1111/irv.12089 24034494 10.1111/irv.12089 PMC5909385

[10] Morens DM, Taubenberger JK, Fauci AS (2008) Predominant role of bacterial pneumonia as a cause of death in pandemic influenza: implications for pandemic influenza preparedness. The Journal of infectious diseases 198: 962–970. doi: 10.1086/591708 18710327 10.1086/591708 PMC2599911

[11] Grassly NC, Fraser C (2008) Mathematical models of infectious disease transmission. Nat Rev Microbiol 6: 477–487. doi: 10.1038/nrmicro1845 18533288 10.1038/nrmicro1845 PMC7097581

[12] Neher RA, Bedford T (2015) nextflu: real-time tracking of seasonal influenza virus evolution in humans. Bioinformatics 31: 3546–3548. doi: 10.1093/bioinformatics/btv381 26115986 10.1093/bioinformatics/btv381 PMC4612219

[13] Viboud C, Bjornstad ON, Smith DL, Simonsen L, Miller MA, et al (2006) Synchrony, waves, and spatial hierarchies in the spread of influenza. Science 312: 447–451. doi: 10.1126/science.1125237 16574822 10.1126/science.1125237

[14] Wu JT, Riley S, Fraser C, Leung GM (2006) Reducing the impact of the next influenza pandemic using household-based public health interventions. PLoS Med 3: e361 doi: 10.1371/journal.pmed.0030361 16881729 10.1371/journal.pmed.0030361 PMC1526768

[15] Longini IM Jr., Halloran ME, Nizam A, Yang Y (2004) Containing pandemic influenza with antiviral agents. Am J Epidemiol 159: 623–633. 15033640 10.1093/aje/kwh092

[16] Gaglani MJ, Piedra PA, Herschler GB, Griffith ME, Kozinetz CA, et al (2004) Direct and total effectiveness of the intranasal, live-attenuated, trivalent cold-adapted influenza virus vaccine against the 2000–2001 influenza A(H1N1) and B epidemic in healthy children. Arch Pediatr Adolesc Med 158: 65–73. doi: 10.1001/archpedi.158.1.65 14706961 10.1001/archpedi.158.1.65

[17] Matrajt L, Halloran ME, Longini IM Jr. (2013) Optimal vaccine allocation for the early mitigation of pandemic influenza. PLoS Comput Biol 9: e1002964 doi: 10.1371/journal.pcbi.1002964 23555207 10.1371/journal.pcbi.1002964 PMC3605056

[18] House T, Baguelin M, Van Hoek AJ, White PJ, Sadique Z, et al (2011) Modelling the impact of local reactive school closures on critical care provision during an influenza pandemic. Proc Biol Sci 278: 2753–2760. doi: 10.1098/rspb.2010.2688 21288945 10.1098/rspb.2010.2688 PMC3145187

[19] Ferguson NM, Cummings DA, Fraser C, Cajka JC, Cooley PC, et al (2006) Strategies for mitigating an influenza pandemic. Nature 442: 448–452. doi: 10.1038/nature04795 16642006 10.1038/nature04795 PMC7095311

[20] Baguelin M, Flasche S, Camacho A, Demiris N, Miller E, et al (2013) Assessing optimal target populations for influenza vaccination programmes: an evidence synthesis and modelling study. PLoS Med 10: e1001527 doi: 10.1371/journal.pmed.1001527 24115913 10.1371/journal.pmed.1001527 PMC3793005

[21] Opatowski L, Fraser C, Griffin J, de Silva E, Van Kerkhove MD, et al (2011) Transmission characteristics of the 2009 H1N1 influenza pandemic: comparison of 8 Southern hemisphere countries. PLoS Pathog 7: e1002225 doi: 10.1371/journal.ppat.1002225 21909272 10.1371/journal.ppat.1002225 PMC3164643

[22] Kucharski AJ, Kwok KO, Wei VW, Cowling BJ, Read JM, et al (2014) The contribution of social behaviour to the transmission of influenza A in a human population. PLoS Pathog 10: e1004206 doi: 10.1371/journal.ppat.1004206 24968312 10.1371/journal.ppat.1004206 PMC4072802

[23] Cauchemez S, Bhattarai A, Marchbanks TL, Fagan RP, Ostroff S, et al (2011) Role of social networks in shaping disease transmission during a community outbreak of 2009 H1N1 pandemic influenza. Proc Natl Acad Sci U S A 108: 2825–2830. doi: 10.1073/pnas.1008895108 21282645 10.1073/pnas.1008895108 PMC3041067

[24] Apolloni A, Poletto C, Colizza V (2013) Age-specific contacts and travel patterns in the spatial spread of 2009 H1N1 influenza pandemic. BMC Infect Dis 13: 176 doi: 10.1186/1471-2334-13-176 23587010 10.1186/1471-2334-13-176 PMC3644502

[25] Shaman J, Pitzer VE, Viboud C, Grenfell BT, Lipsitch M (2010) Absolute humidity and the seasonal onset of influenza in the continental United States. PLoS Biol 8: e1000316 doi: 10.1371/journal.pbio.1000316 20186267 10.1371/journal.pbio.1000316 PMC2826374

[26] Baguelin M, Hoek AJ, Jit M, Flasche S, White PJ, et al (2010) Vaccination against pandemic influenza A/H1N1v in England: a real-time economic evaluation. Vaccine 28: 2370–2384. doi: 10.1016/j.vaccine.2010.01.002 20096762 10.1016/j.vaccine.2010.01.002

[27] Mina MJ, Klugman KP (2014) The role of influenza in the severity and transmission of respiratory bacterial disease. The Lancet Respiratory medicine 2: 750–763. doi: 10.1016/S2213-2600(14)70131-6 25131494 10.1016/S2213-2600(14)70131-6 PMC4823014

[28] Short K, Habets M (2012) Interactions between Streptococcus pneumoniae and influenza virus: a mutually beneficial relationship? Future Microbiology: 609–624. doi: 10.2217/fmb.12.29 22568716 10.2217/fmb.12.29

[29] DaPalma T, Doonan BP, Trager NM, Kasman LM (2010) A systematic approach to virus-virus interactions. Virus research 149: 1–9. doi: 10.1016/j.virusres.2010.01.002 20093154 10.1016/j.virusres.2010.01.002 PMC7172858

[30] Abramson JS, Mills EL, Giebink GS, Quie PG (1982) Depression of monocyte and polymorphonuclear leukocyte oxidative metabolism and bactericidal capacity by influenza A virus. Infection and immunity 35: 350–355. 7054126 10.1128/iai.35.1.350-355.1982 PMC351036

[31] Rynda-Apple A, Robinson KM, Alcorn JF (2015) Influenza and bacterial superinfection: Illuminating the immunologic mechanisms of disease. Infection and Immunity 83: 3764–3770. doi: 10.1128/IAI.00298-15 26216421 10.1128/IAI.00298-15 PMC4567631

[32] Ghoneim HE, Thomas PG, McCullers Ja (2013) Depletion of alveolar macrophages during influenza infection facilitates bacterial superinfections. Journal of immunology (Baltimore, Md: 1950) 191: 1250–1259. 10.4049/jimmunol.1300014 PMC4907362 23804714

[33] Sen GC (2001) Viruses and interferons. Annu Rev Microbiol 55: 255–281. doi: 10.1146/annurev.micro.55.1.255 11544356 10.1146/annurev.micro.55.1.255

[34] Loh E, Kugelberg E, Tracy A, Zhang Q, Gollan B, et al (2013) Temperature triggers immune evasion by Neisseria meningitidis. Nature 502: 237–240. doi: 10.1038/nature12616 24067614 10.1038/nature12616 PMC3836223

[35] Braun LE, Sutter DE, Eichelberger MC, Pletneva L, Kokai-Kun JF, et al (2007) Co-infection of the cotton rat (Sigmodon hispidus) with Staphylococcus aureus and influenza A virus results in synergistic disease. Microbial Pathogenesis 43: 208–216. doi: 10.1016/j.micpath.2007.03.005 17689046 10.1016/j.micpath.2007.03.005

[36] Smith AM, McCullers JA (2014) Secondary bacterial infections in influenza virus infection pathogenesis. Current topics in microbiology and immunology 385: 327–356. doi: 10.1007/82_2014_394 25027822 10.1007/82_2014_394 PMC7122299

[37] Tashiro M, Ciborowski P, Reinacher M, Pulverer G, Klenk HD, et al (1987) Synergistic role of staphylococcal proteases in the induction of influenza virus pathogenicity. Virology 157: 421–430. 3029981 10.1016/0042-6822(87)90284-4

[38] Rohani P, Earn DJ, Finkenstädt B, Grenfell BT (1998) Population dynamic interference among childhood diseases. Proceedings Biological sciences / The Royal Society 265: 2033–2041. 10.1098/rspb.1998.0537 PMC1689490 9842732

[39] Rohani P, Green CJ, Mantilla-Beniers NB, Grenfell BT (2003) Ecological interference between fatal diseases. Nature 422: 885–888. doi: 10.1038/nature01542 12712203 10.1038/nature01542

[40] Turner RD, Bothamley GH (2015) Cough and the transmission of tuberculosis. The Journal of infectious diseases 211: 1367–1372. doi: 10.1093/infdis/jiu625 25387581 10.1093/infdis/jiu625

[41] Cohen R, Varon E, Doit C, Schlemmer C, Romain O, et al (2015) A 13-year survey of pneumococcal nasopharyngeal carriage in children with acute otitis media following PCV7 and PCV13 implementation. Vaccine 33: 5118–5126. doi: 10.1016/j.vaccine.2015.08.010 26271823 10.1016/j.vaccine.2015.08.010

[42] Yildirim I, Little BA, Finkelstein J, Lee G, Hanage WP, et al (2017) Surveillance of pneumococcal colonization and invasive pneumococcal disease reveals shift in prevalent carriage serotypes in Massachusetts' children to relatively low invasiveness. Vaccine 35: 4002–4009. doi: 10.1016/j.vaccine.2017.05.077 28645717 10.1016/j.vaccine.2017.05.077

[43] Barbour ML (1996) Conjugate vaccines and the carriage of Haemophilus influenzae type b. Emerg Infect Dis 2: 176–182. doi: 10.3201/eid0203.960303 8903227 10.3201/eid0203.960303 PMC2626802

[44] Hammitt LL, Crane RJ, Karani A, Mutuku A, Morpeth SC, et al (2016) Effect of Haemophilus influenzae type b vaccination without a booster dose on invasive H influenzae type b disease, nasopharyngeal carriage, and population immunity in Kilifi, Kenya: a 15-year regional surveillance study. Lancet Glob Health 4: e185–194. doi: 10.1016/S2214-109X(15)00316-2 26853149 10.1016/S2214-109X(15)00316-2 PMC4763163

[45] McCullers JA (2006) Insights into the interaction between influenza virus and pneumococcus. Clinical microbiology reviews 19: 571–582. doi: 10.1128/CMR.00058-05 16847087 10.1128/CMR.00058-05 PMC1539103

[46] Zhou H, Haber M, Ray S, Farley MM, Panozzo CA, et al (2012) Invasive pneumococcal pneumonia and respiratory virus co-infections. Emerg Infect Dis 18: 294–297. doi: 10.3201/eid1802.102025 22305270 10.3201/eid1802.102025 PMC3310442

[47] Kim PE, Musher DM, Glezen WP, Rodriguez-Barradas MC, Nahm WK, et al (1996) Association of invasive pneumococcal disease with season, atmospheric conditions, air pollution, and the isolation of respiratory viruses. Clin Infect Dis 22: 100–106. 8824973 10.1093/clinids/22.1.100

[48] Watson M, Gilmour R, Menzies R, Ferson M, McIntyre P, et al (2006) The association of respiratory viruses, temperature, and other climatic parameters with the incidence of invasive pneumococcal disease in Sydney, Australia. Clin Infect Dis 42: 211–215. doi: 10.1086/498897 16355331 10.1086/498897

[49] Toschke AM, Arenz S, von Kries R, Puppe W, Weigl JA, et al (2008) No temporal association between influenza outbreaks and invasive pneumococcal infections. Arch Dis Child 93: 218–220. doi: 10.1136/adc.2006.098996 17405858 10.1136/adc.2006.098996

[50] Sun K, Metzger DW (2014) Influenza Infection Suppresses NADPH Oxidase-Dependent Phagocytic Bacterial Clearance and Enhances Susceptibility to Secondary Methicillin-Resistant Staphylococcus aureus Infection. Journal of immunology (Baltimore, Md: 1950) 192: 3301–3307. 10.4049/jimmunol.1303049 PMC3965630 24563256

[51] Lee LN, Dias P, Han D, Yoon S, Shea A, et al (2010) A mouse model of lethal synergism between influenza virus and Haemophilus influenzae. The American journal of pathology 176: 800–811. doi: 10.2353/ajpath.2010.090596 20042666 10.2353/ajpath.2010.090596 PMC2808086

[52] Hubert B, Watier L, Garnerin P, Richardson S (1992) Meningococcal disease and influenza-like syndrome: a new approach to an old question. The Journal of infectious diseases 166: 542–545. 1500737 10.1093/infdis/166.3.542

[53] Rameix-Welti M-A, Zarantonelli ML, Giorgini D, Ruckly C, Marasescu M, et al (2009) Influenza A virus neuraminidase enhances meningococcal adhesion to epithelial cells through interaction with sialic acid-containing meningococcal capsules. Infection and immunity 77: 3588–3595. doi: 10.1128/IAI.00155-09 19528219 10.1128/IAI.00155-09 PMC2738041

[54] Walaza S, Tempia S, Dawood H, Variava E, Moyes J, et al (2015) Influenza virus infection is associated with increased risk of death amongst patients hospitalized with confirmed pulmonary tuberculosis in South Africa, 2010–2011. BMC Infectious Diseases 15: 26 doi: 10.1186/s12879-015-0746-x 25623944 10.1186/s12879-015-0746-x PMC4316613

[55] Flórido M, Grima Ma, Gillis CM, Xia Y, Turner SJ, et al (2013) Influenza A virus infection impairs mycobacteria-specific T cell responses and mycobacterial clearance in the lung during pulmonary coinfection. J Immunol 191: 302–311. doi: 10.4049/jimmunol.1202824 23698750 10.4049/jimmunol.1202824

[56] Sonoguchi T, Naito H, Hara M, Takeuchi Y, Fukumi H (1985) Cross-Subtype Protection in Humans During Sequential, Overlapping, and/or Concurrent Epidemics Caused by H3N2 and H1N1 Influenza Viruses. Journal of Infectious Diseases 151: 81–88. 3965596 10.1093/infdis/151.1.81

[57] Laurie KL, Guarnaccia TA, Carolan LA, Yan AWC, Aban M, et al (2015) Interval Between Infections and Viral Hierarchy Are Determinants of Viral Interference Following Influenza Virus Infection in a Ferret Model. Journal of Infectious Diseases 212: 1–10. 25943206 10.1093/infdis/jiv260 PMC4633756

[58] Bedford T, Rambaut A, Pascual M (2012) Canalization of the evolutionary trajectory of the human influenza virus. BMC biology 10: 38 doi: 10.1186/1741-7007-10-38 22546494 10.1186/1741-7007-10-38 PMC3373370

[59] Boni MF, Gog JR, Andreasen V, Christiansen FB (2004) Influenza drift and epidemic size: the race between generating and escaping immunity. Theoretical population biology 65: 179–191. doi: 10.1016/j.tpb.2003.10.002 14766191 10.1016/j.tpb.2003.10.002

[60] Smith DJ, Lapedes AS, de Jong JC, Bestebroer TM, Rimmelzwaan GF, et al (2004) Mapping the antigenic and genetic evolution of influenza virus. Science 305: 371–376. doi: 10.1126/science.1097211 15218094 10.1126/science.1097211

[61] Davenport FM, Hennessy AV, Francis T Jr. (1953) Epidemiologic and immunologic significance of age distribution of antibody to antigenic variants of influenza virus. J Exp Med 98: 641–656. 13109114 10.1084/jem.98.6.641 PMC2136340

[62] Gostic KM, Ambrose M, Worobey M, Lloyd-Smith JO (2016) Potent protection against H5N1 and H7N9 influenza via childhood hemagglutinin imprinting. Science 354: 722–726. doi: 10.1126/science.aag1322 27846599 10.1126/science.aag1322 PMC5134739

[63] Kucharski AJ, Gog JR (2012) The role of social contacts and original antigenic sin in shaping the age pattern of immunity to seasonal influenza. PLoS Comput Biol 8: e1002741 doi: 10.1371/journal.pcbi.1002741 23133346 10.1371/journal.pcbi.1002741 PMC3486889

[64] Skowronski DM, De Serres G, Crowcroft NS, Janjua NZ, Boulianne N, et al (2010) Association between the 2008–09 Seasonal Influenza Vaccine and Pandemic H1N1 Illness during Spring–Summer 2009: Four Observational Studies from Canada. PLoS Med 7: e1000258 doi: 10.1371/journal.pmed.1000258 20386731 10.1371/journal.pmed.1000258 PMC2850386

[65] Cowling BJ, Fang VJ, Nishiura H, Chan K-H, Ng S, et al (2012) Increased risk of noninfluenza respiratory virus infections associated with receipt of inactivated influenza vaccine. Clinical infectious diseases: an official publication of the Infectious Diseases Society of America 54: 1778–1783. 22423139 10.1093/cid/cis307 PMC3404712

[66] Casalegno JS, Ottmann M, Duchamp MB, Escuret V, Billaud G, et al (2010) Rhinoviruses delayed the circulation of the pandemic influenza A (H1N1) 2009 virus in France. Clinical microbiology and infection: the official publication of the European Society of Clinical Microbiology and Infectious Diseases 16: 326–329. 10.1111/j.1469-0691.2010.03167.x 20121829

[67] Yang L, Chan KH, Suen LKP, Chan KP, Wang X, et al (2015) Impact of the 2009 H1N1 Pandemic on Age-Specific Epidemic Curves of Other Respiratory Viruses: A Comparison of Pre-Pandemic, Pandemic and Post-Pandemic Periods in a Subtropical City. PLoS ONE 10: e0125447 doi: 10.1371/journal.pone.0125447 25928217 10.1371/journal.pone.0125447 PMC4416050

[68] Navarro-Marí JM, Pérez-Ruiz M, Galán Montemayor JC, Marcos Maeso MÁ, Reina J, et al (2012) Circulation of other respiratory viruses and viral co-infection during the 2009 pandemic influenza. Enfermedades infecciosas y microbiología clínica 30 Suppl 4: 25–31. 23116789 10.1016/S0213-005X(12)70101-5 PMC7130202

[69] Yoshida L-M, Suzuki M, Nguyen HA, Le MN, Dinh Vu T, et al (2013) Respiratory syncytial virus: co-infection and paediatric lower respiratory tract infections. The European respiratory journal 42: 461–469. doi: 10.1183/09031936.00101812 23645407 10.1183/09031936.00101812

[70] Richard N, Komurian-Pradel F, Javouhey E, Perret M, Rajoharison A, et al (2008) The Impact of Dual Viral Infection in Infants Admitted to a Pediatric Intensive Care Unit Associated with Severe Bronchiolitis. The Pediatric Infectious Disease Journal 27: 213–217. doi: 10.1097/INF.0b013e31815b4935 18277932 10.1097/INF.0b013e31815b4935

[71] Marcos MA, Ramón S, Antón A, Martinez E, Vilella A, et al (2011) Clinical relevance of mixed respiratory viral infections in adults with influenza A H1N1. The European respiratory journal 38: 739–742. doi: 10.1183/09031936.00168610 21885422 10.1183/09031936.00168610

[72] Schnepf N, Resche-Rigon M, Chaillon A, Scemla A, Gras G, et al (2011) High burden of non-influenza viruses in influenza-like illness in the early weeks of H1N1v epidemic in France. PLoS ONE 6: e23514 doi: 10.1371/journal.pone.0023514 21858150 10.1371/journal.pone.0023514 PMC3157400

[73] Rhedin S, Hamrin J, Naucler P, Bennet R, Rotzén-Östlund M, et al (2012) Respiratory Viruses in Hospitalized Children with Influenza-Like Illness during the H1n1 2009 Pandemic in Sweden. PLoS ONE 7: e51491 doi: 10.1371/journal.pone.0051491 23272110 10.1371/journal.pone.0051491 PMC3522717

[74] Esper FP, Spahlinger T, Zhou L (2011) Rate and influence of respiratory virus co-infection on pandemic (H1N1) influenza disease. The Journal of infection 63: 260–266. doi: 10.1016/j.jinf.2011.04.004 21546090 10.1016/j.jinf.2011.04.004 PMC3153592

[75] Anestad G (1982) Interference between outbreaks of respiratory syncytial virus and influenza virus infection. Lancet (London, England) 1: 502. 10.1016/s0140-6736(82)91466-0 6121154

[76] Glezen WP, Paredes A, Taber LH (1980) Influenza in children. Relationship to other respiratory agents. JAMA 243: 1345–1349. 6244421 10.1001/jama.243.13.1345

[77] Casalegno JS, Ottmann M, Bouscambert-Duchamp M, Valette M, Morfin F, et al (2010) Impact of the 2009 influenza A(H1N1) pandemic wave on the pattern of hibernal respiratory virus epidemics, France, 2009. Euro surveillance: bulletin Européen sur les maladies transmissibles = European communicable disease bulletin 15: 1. 20158981

[78] Mak GC, Wong AH, Ho WYY, Lim W (2012) The impact of pandemic influenza A (H1N1) 2009 on the circulation of respiratory viruses 2009–2011. Influenza and other respiratory viruses 6: e6–10. doi: 10.1111/j.1750-2659.2011.00323.x 22212717 10.1111/j.1750-2659.2011.00323.x PMC5657134

[79] Yang Y, Wang Z, Ren L, Wang W, Vernet G, et al (2012) Influenza A/H1N1 2009 pandemic and respiratory virus infections, Beijing, 2009–2010. PLoS ONE 7: e45807 doi: 10.1371/journal.pone.0045807 23029253 10.1371/journal.pone.0045807 PMC3447804

[80] Aberle JHMA, Stephan W. MD*; Pracher Elisabeth MD‡; Hutter Hans-Peter MD†; Kundi Michael MD†; Popow-Kraupp Therese MD* (2005) Single Versus Dual Respiratory Virus Infections in Hospitali…: The Pediatric Infectious Disease Journal. The pediatric infectious disease journal 24. 10.1097/01.inf.0000168741.59747.2d 15999001

[81] Goka E, Vallely P, Mutton K, Klapper P (2013) Influenza A viruses dual and multiple infections with other respiratory viruses and risk of hospitalisation and mortality. Influenza Other Respir Viruses 7: 1079–1087. doi: 10.1111/irv.12020 23078095 10.1111/irv.12020 PMC4634299

[82] Walzl G, Tafuro S, Moss P, Openshaw PJ, Hussell T (2000) Influenza virus lung infection protects from respiratory syncytial virus-induced immunopathology. The Journal of experimental medicine 192: 1317–1326. 11067880 10.1084/jem.192.9.1317 PMC2193356

[83] Pascalis H, Temmam S, Turpin M, Rollot O, Flahault A, et al (2012) Intense Co-Circulation of Non-Influenza Respiratory Viruses during the First Wave of Pandemic Influenza pH1N1/2009: A Cohort Study in Reunion Island. PLoS ONE 7: e44755 doi: 10.1371/journal.pone.0044755 22984554 10.1371/journal.pone.0044755 PMC3440351

[84] Greer RM, McErlean P, Arden KE, Faux CE, Nitsche A, et al (2009) Do rhinoviruses reduce the probability of viral co-detection during acute respiratory tract infections? Journal of Clinical Virology 45: 10–15. doi: 10.1016/j.jcv.2009.03.008 19376742 10.1016/j.jcv.2009.03.008 PMC7185458

[85] Murphy BR, Richman DD, Chalhub EG, Uhlendorf CP, Baron S, et al (1975) Failure of attenuated temperature-sensitive influenza A (H3N2) virus to induce heterologous interference in humans to parainfluenza type 1 virus. Infection and immunity 12: 62–68. 166929 10.1128/iai.12.1.62-68.1975 PMC415245

[86] Nisii C, Meschi S, Selleri M, Bordi L, Castilletti C, et al (2010) Frequency of detection of upper respiratory tract viruses in patients tested for pandemic H1N1/09 viral infection. Journal of clinical microbiology 48: 3383–3385. doi: 10.1128/JCM.01179-10 20592147 10.1128/JCM.01179-10 PMC2937695

[87] Goka EA, Vallely PJ, Mutton KJ, Klapper PE (2014) Single and multiple respiratory virus infections and severity of respiratory disease: a systematic review. Paediatric respiratory reviews 15: 363–370. doi: 10.1016/j.prrv.2013.11.001 24361079 10.1016/j.prrv.2013.11.001 PMC7106320

[88] Nicoli EJ, Trotter CL, Turner KME, Colijn C, Waight P, et al (2013) Influenza and RSV make a modest contribution to invasive pneumococcal disease incidence in the UK. The Journal of infection 66: 512–520. doi: 10.1016/j.jinf.2013.02.007 23473714 10.1016/j.jinf.2013.02.007 PMC3650581

[89] Weinberger DM, Harboe ZB, Krause TG, Miller M, Konradsen HB (2013) Serotype-specific effect of influenza on adult invasive pneumococcal pneumonia. Journal of Infectious Diseases: 1–22. 10.1093/infdis/jit375 PMC3888281 23901093

[90] Gilca R, De Serres G, Skowronski D, Boivin G, Buckeridge DL (2009) The need for validation of statistical methods for estimating respiratory virus-attributable hospitalization. Am J Epidemiol 170: 925–936. doi: 10.1093/aje/kwp195 19679751 10.1093/aje/kwp195

[91] Hendriks W, Boshuizen H, Dekkers A, Knol M, Donker GA, et al (2017) Temporal cross-correlation between influenza-like illnesses and invasive pneumococcal disease in The Netherlands. Influenza Other Respir Viruses 11: 130–137. doi: 10.1111/irv.12442 27943624 10.1111/irv.12442 PMC5304567

[92] Upshur RE, Moineddin R, Crighton EJ, Mamdani M (2006) Interactions of viral pathogens on hospital admissions for pneumonia, croup and chronic obstructive pulmonary diseases: results of a multivariate time-series analysis. Epidemiol Infect 134: 1174–1178. doi: 10.1017/S0950268806006236 16623988 10.1017/S0950268806006236 PMC2870510

[93] Randuineau B (2015) Interactions between pathogens: what are the impacts on public health: Universite Pierre et Marie Curie.

[94] Domenech de Celles M, Arduin H, Varon E, Souty C, Boelle PY, et al (2017) Characterizing and Comparing the Seasonality of Influenza-Like Illnesses and Invasive Pneumococcal Diseases Using Seasonal Waveforms. Am J Epidemiol. 10.1093/aje/kwx336 29053767

[95] Boianelli A, Nguyen VK, Ebensen T, Schulze K, Wilk E, et al (2015) Modelling Influenza Virus Infection: A Roadmap for Influenza Research. Viruses 7: 5274–5304. doi: 10.3390/v7102875 26473911 10.3390/v7102875 PMC4632383

[96] Hodgson D, Baguelin M, van Leeuwen E, Panovska-Griffiths J, Ramsay M, et al (2017) Effect of mass paediatric influenza vaccination on existing influenza vaccination programmes in England and Wales: a modelling and cost-effectiveness analysis. Lancet Public Health 2: e74–e81. doi: 10.1016/S2468-2667(16)30044-5 28299371 10.1016/S2468-2667(16)30044-5 PMC5341148

[97] Smith AM, Adler FR, Ribeiro RM, Gutenkunst RN, McAuley JL, et al (2013) Kinetics of coinfection with influenza A virus and Streptococcus pneumoniae. PLoS Pathog 9: e1003238 doi: 10.1371/journal.ppat.1003238 23555251 10.1371/journal.ppat.1003238 PMC3605146

[98] Shrestha S, Foxman B, Dawid S, Aiello AE, Davis BM, et al (2013) Time and dose-dependent risk of pneumococcal pneumonia following influenza: a model for within-host interaction between influenza and Streptococcus pneumoniae. Journal of the Royal Society, Interface / the Royal Society 10: 20130233. 10.1098/rsif.2013.0233 PMC3730679 23825111

[99] Smith AM, Smith AP (2016) A Critical, Nonlinear Threshold Dictates Bacterial Invasion and Initial Kinetics During Influenza. Sci Rep 6: 38703 doi: 10.1038/srep38703 27974820 10.1038/srep38703 PMC5156930

[100] Smith AM (2017) Quantifying the therapeutic requirements and potential for combination therapy to prevent bacterial coinfection during influenza. J Pharmacokinet Pharmacodyn 44: 81–93. doi: 10.1007/s10928-016-9494-9 27679506 10.1007/s10928-016-9494-9 PMC5376398

[101] Boianelli A, Sharma-Chawla N, Bruder D, Hernandez-Vargas EA (2016) Oseltamivir PK/PD Modelling and Simulation to Evaluate Treatment Strategies against Influenza-Pneumococcus Coinfection. Front Cell Infect Microbiol 6: 60 doi: 10.3389/fcimb.2016.00060 27379214 10.3389/fcimb.2016.00060 PMC4906052

[102] Opatowski L, Varon E, Dupont C, Temime L, van der Werf S, et al (2013) Assessing pneumococcal meningitis association with viral respiratory infections and antibiotics: insights from statistical and mathematical models. Proceedings Biological sciences / The Royal Society 280: 20130519. 10.1098/rspb.2013.0519 PMC3712413 23782877

[103] Shrestha S, Foxman B, Berus J, van Panhuis WG, Steiner C, et al (2015) The role of influenza in the epidemiology of pneumonia. Scientific Reports 5: 15314 doi: 10.1038/srep15314 26486591 10.1038/srep15314 PMC4614252

[104] Chien YW, Levin BR, Klugman KP (2012) The anticipated severity of a "1918-like" influenza pandemic in contemporary populations: The contribution of antibacterial interventions. PLoS ONE 7. 10.1371/journal.pone.0029219 PMC3264555 22291887

[105] Crowe S, Utley M, Walker G, Grove P, Pagel C (2011) A model to evaluate mass vaccination against pneumococcus as a countermeasure against pandemic influenza. Vaccine 29: 5065–5077. doi: 10.1016/j.vaccine.2011.04.034 21539879 10.1016/j.vaccine.2011.04.034

[106] Handel A, Longini IM, Antia R (2009) Intervention strategies for an influenza pandemic taking into account secondary bacterial infections. Epidemics 1: 185–195. doi: 10.1016/j.epidem.2009.09.001 20161493 10.1016/j.epidem.2009.09.001 PMC2796779

[107] Shrestha S, Foxman B, Weinberger DM, Steiner C, Viboud C, et al (2013) Identifying the Interaction Between Influenza and Pneumococcal Pneumonia Using Incidence Data. Science Translational Medicine 5: 191ra184–191ra184. 10.1126/scitranslmed.3005982 PMC4178309 23803706

[108] Arduin H, Domenech de Celles M, Guillemot D, Watier L, Opatowski L (2017) An agent-based model simulation of influenza interactions at the host level: insight into the influenza-related burden of pneumococcal infections. BMC Infect Dis 17: 382 doi: 10.1186/s12879-017-2464-z 28577533 10.1186/s12879-017-2464-z PMC5455134

[109] Yan AWC, Cao P, Heffernan JM, McVernon J, Quinn KM, et al (2017) Modelling cross-reactivity and memory in the cellular adaptive immune response to influenza infection in the host. Journal of Theoretical Biology 413: 34–49. doi: 10.1016/j.jtbi.2016.11.008 27856216 10.1016/j.jtbi.2016.11.008

[110] Cao P, Yan AWC, Heffernan JM, Petrie S, Moss RG, et al (2015) Innate Immunity and the Inter-exposure Interval Determine the Dynamics of Secondary Influenza Virus Infection and Explain Observed Viral Hierarchies. PLoS Comput Biol 11: e1004334 doi: 10.1371/journal.pcbi.1004334 26284917 10.1371/journal.pcbi.1004334 PMC4540579

[111] Zakikhany K, Degail MA, Lamagni T, Waight P, Guy R, et al (2011) Increase in invasive streptococcus pyogenes and streptococcus pneumoniae infections in England, December 2010 to January 2011. Eurosurveillance 16: 1–4. 21315057

[112] Pinky L, Dobrovolny HM (2016) Coinfections of the Respiratory Tract: Viral Competition for Resources. PLoS ONE 11: e0155589 doi: 10.1371/journal.pone.0155589 27196110 10.1371/journal.pone.0155589 PMC4873262

[113] Kucharski AJ, Andreasen V, Gog JR (2016) Capturing the dynamics of pathogens with many strains. J Math Biol 72: 1–24. doi: 10.1007/s00285-015-0873-4 25800537 10.1007/s00285-015-0873-4 PMC4698306

[114] Ferguson NM, Galvani AP, Bush RM (2003) Ecological and immunological determinants of influenza evolution. Nature 422: 428–433. doi: 10.1038/nature01509 12660783 10.1038/nature01509

[115] ACKERMAN E, LONGINI IM, SEAHOLM SK, HEDIN ÅS (1990) Simulation of Mechanisms of Viral Interference in Influenza. International Journal of Epidemiology 19: 444–454. 2376460 10.1093/ije/19.2.444

[116] Merler S, Poletti P, Ajelli M, Caprile B, Manfredi P (2008) Coinfection can trigger multiple pandemic waves. Journal of Theoretical Biology 254: 499–507. doi: 10.1016/j.jtbi.2008.06.004 18606170 10.1016/j.jtbi.2008.06.004 PMC7094108

[117] Velasco-Hernández JX, Núñez-López M, Comas-García A, Cherpitel DEN, Ocampo MC, et al (2015) Superinfection between Influenza and RSV Alternating Patterns in San Luis Potosí State, México. PLoS ONE 10: e0115674 doi: 10.1371/journal.pone.0115674 25803450 10.1371/journal.pone.0115674 PMC4372574

[118] Araz OM, Galvani A, Meyers LA (2012) Geographic prioritization of distributing pandemic influenza vaccines. Health Care Management Science 15: 175–187. doi: 10.1007/s10729-012-9199-6 22618029 10.1007/s10729-012-9199-6 PMC4295509

[119] Sa Madhi, Klugman KP (2004) A role for Streptococcus pneumoniae in virus-associated pneumonia. Nature medicine 10: 811–813. doi: 10.1038/nm1077 15247911 10.1038/nm1077 PMC7095883

[120] Simonsen L, Taylor RJ, Young-Xu Y, Haber M, May L, et al (2011) Impact of pneumococcal conjugate vaccination of infants on pneumonia and influenza hospitalization and mortality in all age groups in the United States. MBio 2: e00309–00310. doi: 10.1128/mBio.00309-10 21264063 10.1128/mBio.00309-10 PMC3025524

[121] Tsai Y-H, Hsieh M-J, Chang C-J, Wen Y-W, Hu H-C, et al (2015) The 23-valent pneumococcal polysaccharide vaccine is effective in elderly adults over 75 years old—Taiwan's PPV vaccination program. Vaccine 33: 2897–2902. doi: 10.1016/j.vaccine.2015.04.068 25936662 10.1016/j.vaccine.2015.04.068

[122] Fleming-Dutra KE, Hersh AL, Shapiro DJ, Bartoces M, Enns EA, et al (2016) Prevalence of Inappropriate Antibiotic Prescriptions Among US Ambulatory Care Visits, 2010–2011. JAMA 315: 1864 doi: 10.1001/jama.2016.4151 27139059 10.1001/jama.2016.4151

[123] Polgreen PM, Yang M, Laxminarayan R, Cavanaugh JE (2011) Respiratory fluoroquinolone use and influenza. Infection control and hospital epidemiology 32: 706–709. doi: 10.1086/660859 21666403 10.1086/660859 PMC3258490

[124] Mina MJ, Klugman KP, McCullers JA (2013) Live Attenuated Influenza Vaccine, But Not Pneumococcal Conjugate Vaccine, Protects Against Increased Density and Duration of Pneumococcal Carriage After Influenza Infection in Pneumococcal Colonized Mice. Journal of Infectious Diseases 208: 1281–1285. doi: 10.1093/infdis/jit317 23852122 10.1093/infdis/jit317 PMC6281400

[125] Muthuri SG, Venkatesan S, Myles PR, Leonardi-Bee J, Al Khuwaitir TSA, et al (2014) Effectiveness of neuraminidase inhibitors in reducing mortality in patients admitted to hospital with influenza A H1N1pdm09 virus infection: a meta-analysis of individual participant data. The Lancet Respiratory medicine 2: 395–404. doi: 10.1016/S2213-2600(14)70041-4 24815805 10.1016/S2213-2600(14)70041-4 PMC6637757

[126] Fry AM (2014) Effectiveness of neuraminidase inhibitors for severe influenza. The Lancet Respiratory Medicine. pp. 346–348. doi: 10.1016/S2213-2600(14)70068-2 24815800 10.1016/S2213-2600(14)70068-2

[127] McCullers Ja (2014) The public health policy implications of understanding metabiosis. Cell host & microbe 16: 3–4. 25011101 10.1016/j.chom.2014.06.010

[128] McCullers JA (2011) Preventing and treating secondary bacterial infections with antiviral agents. Antivir Ther 16: 123–135. doi: 10.3851/IMP1730 21447860 10.3851/IMP1730 PMC4907367

[129] McCullers Ja, Rehg JE (2002) Lethal synergism between influenza virus and Streptococcus pneumoniae: characterization of a mouse model and the role of platelet-activating factor receptor. The Journal of infectious diseases 186: 341–350. doi: 10.1086/341462 12134230 10.1086/341462

[130] Andrieu C, Doucet A, Holenstein R (2010) Particle Markov chain Monte Carlo methods. Journal of the Royal Statistical Society Series B-Statistical Methodology 72: 269–342.

[131] King AA, Nguyen D, Ionides EL (2016) Statistical Inference for Partially Observed Markov Processes via the R Package pomp. Journal of Statistical Software 69.

[132] Monto AS (2002) Epidemiology of viral respiratory infections. The American Journal of Medicine 112: 4–12. 11955454 10.1016/S0002-9343(01)01058-0 PMC7172370

[133] Stewart M, Loschen W, Kass-Hout T Enabling ESSENCE to Process and Export Meaningful Use Syndromic Surveillance Data.

[134] Warren-Gash C, Bhaskaran K, Hayward A, Leung GM, Lo S-V, et al (2011) Circulating influenza virus, climatic factors, and acute myocardial infarction: a time series study in England and Wales and Hong Kong. The Journal of infectious diseases 203: 1710–1718. doi: 10.1093/infdis/jir171 21606529 10.1093/infdis/jir171 PMC3100509

[135] Mustaquim D (2014) The Evolution of the WHO/NREVSS Influenza Surveillance System: The Challenges and Opportunities that Accompany Electronic Laboratory Data. Online Journal of Public Health Informatics 6.

[136] Zhao H, Green H, Lackenby A, Donati M, Ellis J, et al (2014) A new laboratory-based surveillance system (Respiratory DataMart System) for influenza and other respiratory viruses in England: results and experience from 2009 to 2012. Eurosurveillance 19: 20680 24480060 10.2807/1560-7917.es2014.19.3.20680

[137] Wolf AI, Strauman MC, Mozdzanowska K, Whittle JRR, Williams KL, et al (2014) Coinfection with Streptococcus pneumoniae Modulates the B Cell Response to Influenza Virus. Journal of Virology 88: 11995–12005. doi: 10.1128/JVI.01833-14 25100838 10.1128/JVI.01833-14 PMC4178749

[138] Siegel Steven J, Roche Aoife M, Weiser Jeffrey N (2014) Influenza Promotes Pneumococcal Growth during Coinfection by Providing Host Sialylated Substrates as a Nutrient Source. Cell Host & Microbe 16: 55–67. 25011108 10.1016/j.chom.2014.06.005 PMC4096718

[139] McCullers Ja, McAuley JL, Browall S, Iverson AR, Boyd KL, et al (2010) Influenza enhances susceptibility to natural acquisition of and disease due to Streptococcus pneumoniae in ferrets. The Journal of infectious diseases 202: 1287–1295. doi: 10.1086/656333 20822454 10.1086/656333 PMC3249639

[140] Peltola VT, Boyd KL, McAuley JL, Rehg JE, McCullers JA (2006) Bacterial sinusitis and otitis media following influenza virus infection in ferrets. Infection and immunity 74: 2562–2567. doi: 10.1128/IAI.74.5.2562-2567.2006 16622191 10.1128/IAI.74.5.2562-2567.2006 PMC1459735

[141] Walters K-A, D'Agnillo F, Sheng Z-M, Kindrachuk J, Schwartzman LM, et al (2016) 1918 pandemic influenza virus and Streptococcus pneumoniae co-infection results in activation of coagulation and widespread pulmonary thrombosis in mice and humans. The Journal of Pathology 238: 85–97. doi: 10.1002/path.4638 26383585 10.1002/path.4638 PMC4789761

[142] Nakamura S, Davis KM, Weiser JN, Bogaert D, Groot RD, et al (2011) Synergistic stimulation of type I interferons during influenza virus coinfection promotes Streptococcus pneumoniae colonization in mice. Journal of Clinical Investigation 121: 3657–3665. doi: 10.1172/JCI57762 21841308 10.1172/JCI57762 PMC3163966

[143] Walter ND, Taylor TH, Shay DK, Thompson WW, Brammer L, et al (2010) Influenza circulation and the burden of invasive pneumococcal pneumonia during a non-pandemic period in the United States. Clinical infectious diseases: an official publication of the Infectious Diseases Society of America 50: 175–183. 20014948 10.1086/649208

[144] Nelson GE, Gershman Ka, Swerdlow DL, Beall BW, Moore MR (2012) Invasive pneumococcal disease and pandemic (H1N1) 2009, Denver, Colorado, USA. Emerging infectious diseases 18: 208–216. doi: 10.3201/eid1802.110714 22306234 10.3201/eid1802.110714 PMC3310448

[145] Jansen AGSC, Sanders EAM, VAN DER Ende A, VAN Loon AM, Hoes AW, et al (2008) Invasive pneumococcal and meningococcal disease: association with influenza virus and respiratory syncytial virus activity? Epidemiology and infection 136: 1448–1454. doi: 10.1017/S0950268807000271 18211724 10.1017/S0950268807000271 PMC2870742

[146] Kuster SP, Tuite AR, Kwong JC, McGeer A, Fisman DN (2011) Evaluation of coseasonality of influenza and invasive pneumococcal disease: results from prospective surveillance. PLoS Med 8: e1001042 doi: 10.1371/journal.pmed.1001042 21687693 10.1371/journal.pmed.1001042 PMC3110256

[147] Ampofo K, Bender J, Sheng X, Korgenski K, Daly J, et al (2008) Seasonal invasive pneumococcal disease in children: role of preceding respiratory viral infection. Pediatrics 122: 229–237. doi: 10.1542/peds.2007-3192 18676537 10.1542/peds.2007-3192

[148] Grabowska K, Hogberg L, Penttinen P, Svensson A, Ekdahl K (2006) Occurrence of invasive pneumococcal disease and number of excess cases due to influenza. BMC Infect Dis 6: 58 doi: 10.1186/1471-2334-6-58 16549029 10.1186/1471-2334-6-58 PMC1534049

[149] Murdoch DR, Jennings LC (2009) Association of respiratory virus activity and environmental factors with the incidence of invasive pneumococcal disease. J Infect 58: 37–46. doi: 10.1016/j.jinf.2008.10.011 19042025 10.1016/j.jinf.2008.10.011

[150] Edwards LJ, Markey PG, Cook HM, Trauer JM, Krause VL (2011) The relationship between influenza and invasive pneumococcal disease in the Northern Territory, 2005–2009. Med J Aust 194: 207 21401468 10.5694/j.1326-5377.2011.tb03779.x

[151] Weinberger DM, Harboe ZB, Viboud C, Krause TG, Miller M, et al (2014) Pneumococcal disease seasonality: incidence, severity and the role of influenza activity. Eur Respir J 43: 833–841. doi: 10.1183/09031936.00056813 24036243 10.1183/09031936.00056813

[152] Grijalva CG, Griffin MR, Edwards KM, Williams JV, Gil AI, et al (2014) The role of influenza and parainfluenza infections in nasopharyngeal pneumococcal acquisition among young children. Clin Infect Dis 58: 1369–1376. doi: 10.1093/cid/ciu148 24621951 10.1093/cid/ciu148 PMC4001292

[153] Zhou H, Haber M, Ray S, Farley MM, Panozzo CA, et al (2012) Invasive pneumococcal pneumonia and respiratory virus co-infections. Emerging infectious diseases 18: 294–297. doi: 10.3201/eid1802.102025 22305270 10.3201/eid1802.102025 PMC3310442

[154] Damasio GAC, Pereira LA, Moreira SDR, Duarte dos Santos CN, Dalla-Costa LM, et al (2015) Does virus-bacteria coinfection increase the clinical severity of acute respiratory infection? Journal of Medical Virology 87: 1456–1461. doi: 10.1002/jmv.24210 25976175 10.1002/jmv.24210 PMC7166438

[155] Niemann S, Ehrhardt C, Medina E, Warnking K, Tuchscherr L, et al (2012) Combined action of influenza virus and Staphylococcus aureus panton-valentine leukocidin provokes severe lung epithelium damage. The Journal of infectious diseases 206: 1138–1148. doi: 10.1093/infdis/jis468 22837490 10.1093/infdis/jis468 PMC3433859

[156] Davison VE, Sanford BA (1982) Factors influencing adherence of Staphylococcus aureus to influenza A virus-infected cell cultures. Infection and Immunity 37: 946–955. 6813268 10.1128/iai.37.3.946-955.1982 PMC347630

[157] Zhang WJ, Sarawar S, Nguyen P, Daly K, Rehg JE, et al (1996) Lethal synergism between influenza infection and staphylococcal enterotoxin B in mice. J Immunol 157: 5049–5060. 8943414

[158] Chertow DS, Kindrachuk J, Sheng Z-M, Pujanauski LM, Cooper K, et al (2016) Influenza A and Methicillin-resistant Staphylococcus aureus Co-infection in Rhesus Macaques ‐‐ A Model of Severe Pneumonia. Antiviral Research. 10.1016/j.antiviral.2016.02.013 PMC6617511 26923881

[159] Iverson AR, Boyd KL, McAuley JL, Plano LR, Hart ME, et al (2011) Influenza virus primes mice for pneumonia from Staphylococcus aureus. The Journal of infectious diseases 203: 880–888. doi: 10.1093/infdis/jiq113 21278211 10.1093/infdis/jiq113 PMC3071123

[160] Robinson KM, Choi SM, McHugh KJ, Mandalapu S, Enelow RI, et al (2013) Influenza A Exacerbates Staphylococcus aureus Pneumonia by Attenuating IL-1 Production in Mice. The Journal of Immunology 191: 5153–5159. doi: 10.4049/jimmunol.1301237 24089191 10.4049/jimmunol.1301237 PMC3827735

[161] Sherertz RJ, Reagan DR, Hampton KD, Robertson KL, Streed SA, et al (1996) A cloud adult: the Staphylococcus aureus-virus interaction revisited. Ann Intern Med 124: 539–547. 8597316 10.7326/0003-4819-124-6-199603150-00001

[162] Hageman JC, Uyeki TM, Francis JS, Jernigan DB, Wheeler JG, et al (2006) Severe community-acquired pneumonia due to Staphylococcus aureus, 2003–04 influenza season. Emerging infectious diseases 12: 894–899. doi: 10.3201/eid1206.051141 16707043 10.3201/eid1206.051141 PMC3373026

[163] Finelli L, Fiore A, Dhara R, Brammer L, Shay DK, et al (2008) Influenza-associated pediatric mortality in the United States: increase of Staphylococcus aureus coinfection. Pediatrics 122: 805–811. doi: 10.1542/peds.2008-1336 18829805 10.1542/peds.2008-1336

[164] Reed C, Kallen AJ, Patton M, Arnold KE, Farley MM, et al (2009) Infection With Community-Onset Staphylococcus aureus and Influenza Virus in Hospitalized Children. The Pediatric Infectious Disease Journal 28: 572–576. doi: 10.1097/INF.0b013e31819d8b71 19478685 10.1097/INF.0b013e31819d8b71

[165] Kobayashi SD, Olsen RJ, LaCasse RA, Safronetz D, Ashraf M, et al (2013) Seasonal H3N2 influenza A virus fails to enhance Staphylococcus aureus co-infection in a non-human primate respiratory tract infection model. Virulence 4: 707–715. doi: 10.4161/viru.26572 24104465 10.4161/viru.26572 PMC3925702

[166] Michaels RH, Myerowitz RL, Klaw R (1977) Potentiation of experimental meningitis due to Haemophilus influenzae by influenza A virus. The Journal of infectious diseases 135: 641–645. 300760 10.1093/infdis/135.4.641

[167] Bakaletz LO, Hoepf TM, Demaria TF, Lim DJ (1988) The Effect of Antecedent Influenza A Virus Infection on the Adherence of Hemophilus Influenzae to Chinchilla Tracheal Epithelium. Am ~ Otalaryngol 9: 127–134. 10.1016/s0196-0709(88)80018-8 2845827

[168] Francis T, De Torregrosa MV (1945) Combined infection of mice with H. Influenzae and influenza virus by the intranasal route. Journal of Infectious Diseases 76: 70–77.

[169] Read RC, Goodwin L, Parsons MA, Silcocks P, Kaczmarski EB, et al (1999) Coinfection with influenza B virus does not affect association of Neisseria meningitidis with human nasopharyngeal mucosa in organ culture. Infection and immunity 67: 3082–3086. 10338524 10.1128/iai.67.6.3082-3086.1999 PMC96625

[170] Cartwright KA, Jones DM, Smith AJ, Stuart JM, Kaczmarski EB, et al (1991) Influenza A and meningococcal disease. Lancet (London, England) 338: 554–557. 10.1016/0140-6736(91)91112-8 1678811

[171] Jacobs JH, Viboud C, Tchetgen ET, Schwartz J, Steiner C, et al (2014) The Association of Meningococcal Disease with Influenza in the United States, 1989–2009. PLoS ONE 9: e107486 doi: 10.1371/journal.pone.0107486 25265409 10.1371/journal.pone.0107486 PMC4180274

[172] Brundage JF (2006) Interactions between influenza and bacterial respiratory pathogens: implications for pandemic preparedness. The Lancet Infectious diseases 6: 303–312. doi: 10.1016/S1473-3099(06)70466-2 16631551 10.1016/S1473-3099(06)70466-2 PMC7106411

[173] Makras P, Alexiou-Daniel S, Antoniadis A, Hatzigeorgiou D (2001) Outbreak of meningococcal disease after an influenza B epidemic at a Hellenic Air Force recruit training center. Clinical infectious diseases: an official publication of the Infectious Diseases Society of America 33: e48–50. 11512107 10.1086/322609

[174] Florido M, Pillay R, Gillis CM, Xia Y, Turner SJ, et al (2015) Epitope-specific CD4+, but not CD8+, T-cell responses induced by recombinant influenza A viruses protect against Mycobacterium tuberculosis infection. European Journal of Immunology 45: 780–793. doi: 10.1002/eji.201444954 25430701 10.1002/eji.201444954

[175] Volkert M, Pierce C, Horsfall FL, Dubos RJ (1947) THE ENHANCING EFFECT OF CONCURRENT INFECTION WITH PNEUMOTROPIC VIRUSES ON PULMONARY TUBERCULOSIS IN MICE. The Journal of experimental medicine 86: 203–214. 19871671 10.1084/jem.86.3.203 PMC2135728

[176] Redford PS, Mayer-Barber KD, McNab FW, Stavropoulos E, Wack A, et al (2014) Influenza A virus impairs control of Mycobacterium tuberculosis coinfection through a type I interferon receptor-dependent pathway. The Journal of infectious diseases 209: 270–274. doi: 10.1093/infdis/jit424 23935205 10.1093/infdis/jit424 PMC3873785

[177] Walaza S, Cohen C, Nanoo A, Cohen AL, McAnerney J, et al (2015) Excess mortality associated with influenza among tuberculosis deaths in South Africa, 1999–2009. PLoS ONE 10: 1999–2009. 10.1371/journal.pone.0129173 PMC4467974 26076197

[178] Oei W, Nishiura H, Oei W, Nishiura H (2012) The Relationship between Tuberculosis and Influenza Death during the Influenza (H1N1) Pandemic from 1918–19. Computational and Mathematical Methods in Medicine 2012: 1–9. 10.1155/2012/124861 PMC3405656 22848231

[179] Noymer A (2011) The 1918 influenza pandemic hastened the decline of tuberculosis in the United States: An age, period, cohort analysis. Vaccine 29: B38–B41. doi: 10.1016/j.vaccine.2011.02.053 21757102 10.1016/j.vaccine.2011.02.053 PMC3139993

[180] Noymer A (2009) Testing the influenza–tuberculosis selective mortality hypothesis with Union Army data. Social Science & Medicine 68: 1599–1608. 19304361 10.1016/j.socscimed.2009.02.021 PMC2677170

[181] Zürcher K, Zwahlen M, Ballif M, Rieder HL, Egger M, et al (2016) Influenza Pandemics and Tuberculosis Mortality in 1889 and 1918: Analysis of Historical Data from Switzerland. PLoS ONE 11: e0162575 doi: 10.1371/journal.pone.0162575 27706149 10.1371/journal.pone.0162575 PMC5051959

[182] Roth S, Whitehead S, Thamthitiwat S, Chittaganpitch M, Maloney SA, et al (2013) Concurrent influenza virus infection and tuberculosis in patients hospitalized with respiratory illness in Thailand. Influenza and other Respiratory Viruses 7: 244–248. doi: 10.1111/j.1750-2659.2012.00413.x 22817684 10.1111/j.1750-2659.2012.00413.x PMC5779833

[183] Klonoski JM, Hurtig HR, Juber BA, Schuneman MJ, Bickett TE, et al (2014) Vaccination against the M protein of Streptococcus pyogenes prevents death after influenza virus:S. pyogenes super-infection. Vaccine 32: 5241–5249. doi: 10.1016/j.vaccine.2014.06.093 25077423 10.1016/j.vaccine.2014.06.093 PMC4146501

[184] Okamoto S, Kawabata S, Nakagawa I, Okuno Y, Goto T, et al (2003) Influenza A Virus-Infected Hosts Boost an Invasive Type of Streptococcus pyogenes Infection in Mice. Journal of Virology 77: 4104–4112. doi: 10.1128/JVI.77.7.4104-4112.2003 12634369 10.1128/JVI.77.7.4104-4112.2003 PMC150641

[185] Okamoto S, Kawabata S, Terao Y, Fujitaka H, Okuno Y, et al (2004) The Streptococcus pyogenes Capsule Is Required for Adhesion of Bacteria to Virus-Infected Alveolar Epithelial Cells and Lethal Bacterial-Viral Superinfection. Infection and Immunity 72: 6068–6075. doi: 10.1128/IAI.72.10.6068-6075.2004 15385511 10.1128/IAI.72.10.6068-6075.2004 PMC517596

[186] Hafez MM, Abdel-Wahab KSE, El-Fouhil DFI (2010) Augmented adherence and internalization of group A Streptococcus pyogenes to influenza A virus infected MDCK cells. Journal of Basic Microbiology 50: S46–S57. doi: 10.1002/jobm.200900427 20967785 10.1002/jobm.200900427

[187] Scaber J, Saeed S, Ihekweazu C, Efstratiou A, Mccarthy N, et al (2011) Group A streptococcal infections during the seasonal influenza outbreak 2010/11 in South East England. Euro Surveill 16. 21315058

[188] Tasher D, Stein M, Simões EAF, Shohat T, Bromberg M, et al (2011) Invasive bacterial infections in relation to influenza outbreaks, 2006–2010. Clinical infectious diseases: an official publication of the Infectious Diseases Society of America 53: 1199–1207. 22021918 10.1093/cid/cir726

[189] Tamayo E, Montes M, Vicente D, Pérez-Trallero E, Welch C, et al (2016) Streptococcus pyogenes Pneumonia in Adults: Clinical Presentation and Molecular Characterization of Isolates 2006–2015. PLoS ONE 11: e0152640 doi: 10.1371/journal.pone.0152640 27027618 10.1371/journal.pone.0152640 PMC4814053

[190] Anestad G, Vainio K, Hungnes O (2007) Interference between outbreaks of epidemic viruses. Scandinavian journal of infectious diseases 39: 653–654. doi: 10.1080/00365540701253860 17577842 10.1080/00365540701253860

[191] Ånestad G (2009) Surveillance of respiratory viral infections by rapid immunofluorescence diagnosis, with emphasis on virus interference. Epidemiology and Infection 99: 523. 10.1017/s0950268800068023 PMC2249295 2824225

[192] Anestad G (1987) Surveillance of respiratory viral infections by rapid immunofluorescence diagnosis, with emphasis on virus interference. Epidemiol Infect 99: 523–531. 2824225 10.1017/s0950268800068023 PMC2249295

[193] Nishimura N, Nishio H, Lee MJ, Uemura K (2005) The clinical features of respiratory syncytial virus: lower respiratory tract infection after upper respiratory tract infection due to influenza virus. Pediatr Int 47: 412–416. doi: 10.1111/j.1442-200x.2005.02099.x 16091079 10.1111/j.1442-200x.2005.02099.x

[194] van Asten L, Bijkerk P, Fanoy E, van Ginkel A, Suijkerbuijk A, et al (2016) Early occurrence of influenza A epidemics coincided with changes in occurrence of other respiratory virus infections. Influenza and other respiratory viruses 10: 14–26. doi: 10.1111/irv.12348 26369646 10.1111/irv.12348 PMC4687500

[195] Meningher T, Hindiyeh M, Regev L, Sherbany H, Mendelson E, et al (2014) Relationships between A(H1N1)pdm09 influenza infection and infections with other respiratory viruses. Influenza and other respiratory viruses 8: 422–430. doi: 10.1111/irv.12249 24698156 10.1111/irv.12249 PMC4181801

[196] Martin ET, Fairchok MP, Stednick ZJ, Kuypers J, Englund JA (2013) Epidemiology of multiple respiratory viruses in childcare attendees. The Journal of infectious diseases 207: 982–989. doi: 10.1093/infdis/jis934 23288925 10.1093/infdis/jis934 PMC7107308

[197] Shinjoh M, Omoe K, Saito N, Matsuo N, Nerome K (2000) In vitro growth profiles of respiratory syncytial virus in the presence of influenza virus. Acta Virol 44: 91–97. 10989700

[198] Linde A, Rotzen-Ostlund M, Zweygberg-Wirgart B, Rubinova S, Brytting M (2009) Does viral interference affect spread of influenza? Euro Surveill 14. 19822124

[199] Anestad G, Nordbo SA (2011) Virus interference. Did rhinoviruses activity hamper the progress of the 2009 influenza A (H1N1) pandemic in Norway? Med Hypotheses 77: 1132–1134. doi: 10.1016/j.mehy.2011.09.021 21975051 10.1016/j.mehy.2011.09.021

[200] Tanner H, Boxall E, Osman H (2012) Respiratory viral infections during the 2009–2010 winter season in Central England, UK: incidence and patterns of multiple virus co-infections. European journal of clinical microbiology & infectious diseases: official publication of the European Society of Clinical Microbiology 31: 3001–3006. 10.1007/s10096-012-1653-3 PMC7088042 22678349

[201] Mackay IM, Lambert SB, Faux CE, Arden KE, Nissen MD, et al (2013) Community-wide, contemporaneous circulation of a broad spectrum of human rhinoviruses in healthy Australian preschool-aged children during a 12-month period. The Journal of infectious diseases 207: 1433–1441. doi: 10.1093/infdis/jis476 22829638 10.1093/infdis/jis476 PMC7107377

[202] Easton AJ, Scott PD, Edworthy NL, Meng B, Marriott AC, et al (2011) A novel broad-spectrum treatment for respiratory virus infections: influenza-based defective interfering virus provides protection against pneumovirus infection in vivo. Vaccine 29: 2777–2784. doi: 10.1016/j.vaccine.2011.01.102 21320545 10.1016/j.vaccine.2011.01.102

[203] Goto H, Ihira H, Morishita K, Tsuchiya M, Ohta K, et al (2016) Enhanced growth of influenza A virus by coinfection with human parainfluenza virus type 2. Med Microbiol Immunol 205: 209–218. doi: 10.1007/s00430-015-0441-y 26582554 10.1007/s00430-015-0441-y PMC7086786

