# Supplementary figures and images for: Influenza interaction with cocirculating pathogens and its impact on surveillance, pathogenesis, and epidemic profile: A key role for mathematical modelling
Source: PLoS Pathog. 2018 Feb 15;14(2):e1006770. doi: 10.1371/journal.ppat.1006770 (PMC5814058; doi:10.1371/journal.ppat.1006770)

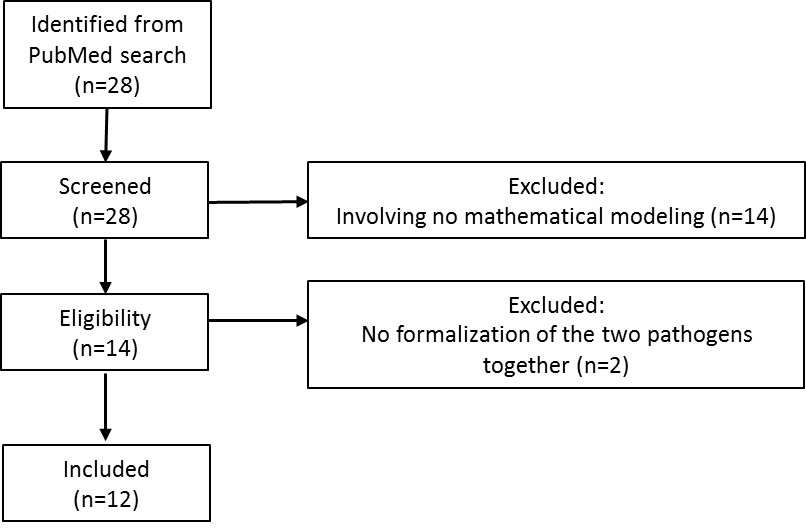

Supplement: S1 Fig — (TIF) [file ppat.1006770.s001.tif]

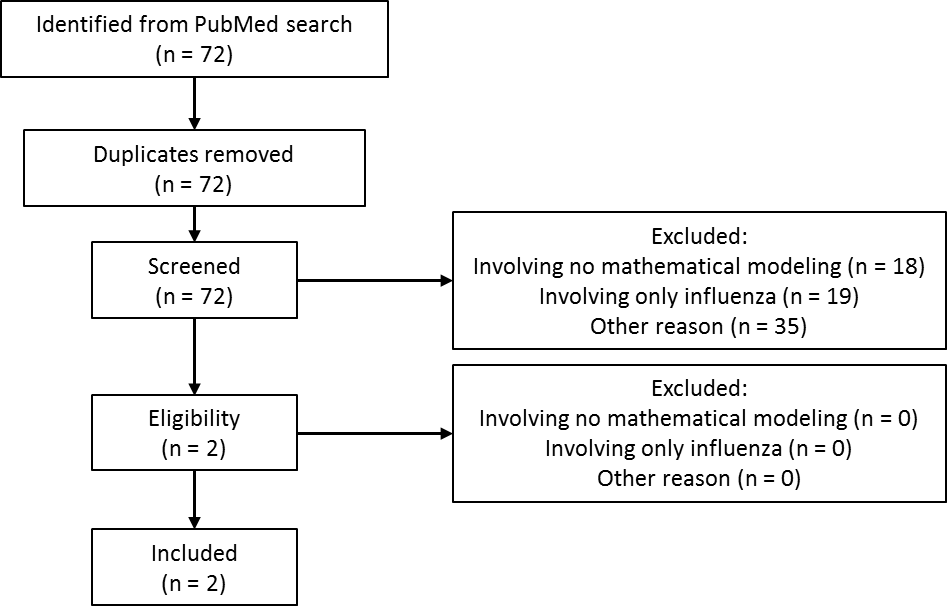

Supplement: S2 Fig — (TIF) [file ppat.1006770.s002.tif]
