# Supplementary material for: Influenza interaction with cocirculating pathogens and its impact on surveillance, pathogenesis, and epidemic profile: A key role for mathematical modelling
Source: PLoS Pathog. 2018 Feb 15;14(2):e1006770. doi: 10.1371/journal.ppat.1006770 (PMC5814058; doi:10.1371/journal.ppat.1006770)
Supplement: S1 Appendix — (DOCX) [file ppat.1006770.s003.docx]

**S1- Appendix to “Influenza Interaction with Cocirculating Pathogens, and Its Impact on Surveillance, Pathogenesis and Epidemic Profile:**

**A Key Role for Mathematical Modeling”**

Lulla Opatowski*, Marc Baguelin, Rosalind M Eggo*.

1. **Search strategy**
2. **Evidence of interaction**

Here, we aimed to gather examples of research investigating influenza interactions. Due to the large number of pathogens covered by the article and consequently the large number of studies, we did not carry out a systematic review but rather our selection aimed at covering both studies concluding in a significant association and studies finding no significant signal of interaction.

For each pathogen, articles were selected based on an independent search in PubMed and bibliographies of the selected articles. The applied algorithm was the following:

For bacteria: (bacteria name) AND (influenza OR flu) AND (coinfection OR co-infection OR interaction OR interference OR superinfection OR association OR complication*)

For viruses: (virus name) AND (influenza OR flu) AND (coinfection OR co-infection OR interaction OR interference OR superinfection OR association OR complication*)

1. **Mathematical models**

We carried out a systematic review of the published modeling studies that investigated influenza interactions.

The search algorithm was as follows:

**Search for influenza-bacteria models:**

("mathematical model*" OR "dynamic model" OR "mechanistic" OR "kinetic" OR "within-host model" OR "population model" OR "individual based" OR "simulation") AND (influenza OR flu) AND (bacteria* OR pneumococc* OR staphylococc* OR influenzae* OR meningitidis OR tuberculosis OR pyogenes) AND (coinfection OR co-infection OR interaction OR interference OR superinfection OR association OR complication*)

Run in PubMed on June 1rst, 2017.

Screened

(n=28)

Eligibility

(n=14)

Excluded:

Involving no mathematical modeling (n=14)

Excluded:

No formalization of the two pathogens together (n=2)

Included

(n=12)

Identified from PubMed search

(n=28)

Figure S1. PRISMA diagram for search for virus-bacteria interactions between influenza and bacteria.

**Search for influenza-virus models:**

("mathematical model*" OR "dynamic model" OR "mechanistic" OR "kinetic" OR "within-host model" OR "population model" OR "individual based" OR "simulation" OR “transmiss*”) AND (influenza OR flu) AND (rhinovirus OR HRV OR parainfluenza OR HPIV OR RSV OR respiratory syncytial OR strain*) AND (coinfection OR co-infection OR interaction OR interference OR superinfection OR association OR complication*)

Run in PubMed on July 31 2017.

Identified from PubMed search

(n = 72)

Duplicates removed

(n = 72)

Excluded:

Involving no mathematical modeling (n = 18)

Involving only influenza (n = 19)

Other reason (n = 35)

Screened

(n = 72)

Eligibility

(n = 2)

Excluded:

Involving no mathematical modeling (n = 0)

Involving only influenza (n = 0)

Other reason (n = 0)

Included

(n = 2)

Figure S2. PRISMA diagram for search for virus-virus interactions between influenza and other viruses. Other reasons include no interaction actually studied focus on influenza and pneumococcus, or study of antiviral pharmaceuticals.

1. **A model for coinfection**

The model shown in the main text can be used for 2 types of interactions:

1. Altering the infectiousness of coinfected individuals, which is controlled by the σ parameters. Values less than 1 decrease infectiousness of coinfected individuals, and greater than 1 increase it. σ_1_ is the effect of pathogen 1 on pathogen 2, so if σ_1_ is less than 1 then pathogen 2 is less infectious in the presence of pathogen 1.
2. Altering the probability of getting a second infection when an individual is already infected, which is controlled by the δ parameters. Values less than 1 decrease the probability of coinfection, and values greater than 1 increase it. δ_1_ is the effect of pathogen 1 on pathogen 2, so when δ_1_ is less than 1, the probability of someone infected with pathogen 1 getting pathogen 2 is decreased.

**1. Equations**

$$\frac{dS}{dt}=-\lambda_{1}S-\lambda_{2}S$$

$$\frac{dI_{1}}{dt}=+\lambda_{1}S-\lambda_{12}I_{1}-\gamma_{1}I_{1}$$

$$\frac{dI_{2}}{dt}=+\lambda_{2}S-\lambda_{21}I_{2}-\gamma_{2}I_{2}$$

$$\frac{dI_{12}}{dt}=+\lambda_{12}I_{1}-\gamma_{12}I_{12}$$

$$\frac{dI_{21}}{dt}=+\lambda_{21}I_{2}-\gamma_{21}I_{21}$$

$$\frac{dC_{12}}{dt}=+\lambda_{2}R_{1}-\gamma_{2}C_{12}$$

$$\frac{dC_{21}}{dt}=+\lambda_{1}R_{2}-\gamma_{1}C_{21}$$

$$\frac{dR_{1}}{dt}=+\gamma_{1}I_{1}-\lambda_{2}R_{1}$$

$$\frac{dR_{2}}{dt}=+\gamma_{2}I_{2}-\lambda_{1}R_{2}$$

$$\frac{dR_{12}}{dt}=+\gamma_{12}I_{12}+\gamma_{2}C_{12}$$

$$\frac{dR_{21}}{dt}=+\gamma_{21}I_{21}+\gamma_{1}C_{21}$$

$$\lambda_{1}=\beta_{1}\left( \frac{I_{1}}{N}+\sigma_{2}\frac{I_{12}}{N}+\sigma_{2}\frac{I_{21}}{N}+\frac{C_{21}}{N} \right)$$

$$\lambda_{21}=\delta_{2}\beta_{1}\left( \frac{I_{1}}{N}+\sigma_{2}\frac{I_{12}}{N}+\sigma_{2}\frac{I_{21}}{N}+\frac{C_{21}}{N} \right)$$

$$\lambda_{2}=\beta_{2}\left( \frac{I_{2}}{N}+\sigma_{1}\frac{I_{12}}{N}+\sigma_{1}\frac{I_{21}}{N}+\frac{C_{12}}{N} \right)$$

$$\lambda_{12}=\delta_{1}\beta_{2}\left( \frac{I_{2}}{N}+\sigma_{1}\frac{I_{12}}{N}+\sigma_{1}\frac{I_{21}}{N}+\frac{C_{12}}{N} \right)$$
